# Gene-Based Mapping and Pathway Analysis of Metabolic Traits in Dairy Cows

**DOI:** 10.1371/journal.pone.0122325

**Published:** 2015-03-19

**Authors:** Ngoc-Thuy Ha, Josef Johann Gross, Annette van Dorland, Jens Tetens, Georg Thaller, Martin Schlather, Rupert Bruckmaier, Henner Simianer

**Affiliations:** 1 Veterinary Physiology, Vetsuisse Faculty, University of Bern, Bern, Switzerland; 2 Animal Breeding and Genetics Group, Department of Animal Sciences, Georg-August-University, Goettingen, Germany; 3 Institute of Animal Breeding and Husbandry, Christian-Albrechts-University, Kiel, Germany; 4 Chair of Mathematical Statistics, University of Mannheim, Mannheim, Germany; Justus-Liebig-Universität, GERMANY

## Abstract

The metabolic adaptation of dairy cows during the transition period has been studied intensively in the last decades. However, until now, only few studies have paid attention to the genetic aspects of this process. Here, we present the results of a gene-based mapping and pathway analysis with the measurements of three key metabolites, (1) non-esterified fatty acids (NEFA), (2) beta-hydroxybutyrate (BHBA) and (3) glucose, characterizing the metabolic adaptability of dairy cows before and after calving. In contrast to the conventional single-marker approach, we identify 99 significant and biologically sensible genes associated with at least one of the considered phenotypes and thus giving evidence for a genetic basis of the metabolic adaptability. Moreover, our results strongly suggest three pathways involved in the metabolism of steroids and lipids are potential candidates for the adaptive regulation of dairy cows in their early lactation. From our perspective, a closer investigation of our findings will lead to a step forward in understanding the variability in the metabolic adaptability of dairy cows in their early lactation.

## Introduction

Selective breeding of dairy cows during the last decades has led to the modern high-yielding dairy cow producing more than 45 kg milk per day [1]. However, the immense milk yield also entails a high energy demand during the early lactation period, which cannot be fully covered by feed intake [2]. In order to overcome the metabolic load resulting from a negative energy balance, dairy cows need to mobilize body fat, protein and mineral stores. A failure in metabolic adaptation to this situation leads to an increased susceptibility to health problems as well as development of production-related diseases such as ketosis and fatty liver [3,4].

Numerous studies have tried to elucidate and describe the complex system of metabolic adaptation of dairy cows during their early lactation period [2,5–9]. They identified a number of crucial regulated target genes, metabolites and endocrine factors in the liver and blood plasma that are involved in important pathways responsible for the adaptive regulation of the metabolism. Nevertheless, so far, a general understanding of why and how the ability of adaptation varies between cows has still not been reached.

Interestingly, even under the same conditions and similar production levels, the success of adaptation differs substantially between cows [5,6]. This strongly suggests that the ability of adaptation may have a genetic basis. In this study, we aim to identify the genetic factors influencing the metabolic adaptation performance during early lactation. In particular, we are interested in identifying genes as well as pathways associated with levels of candidate metabolites in the blood plasma, which were previously confirmed to be essentially involved in the regulation of metabolic adaptation in dairy cows [2,4,6–10].

Genome-wide association studies (GWAS) using single marker analysis (SMA) have been an essential tool for identifying genetic effects. GWAS approaches have been used to detect genomic regions which affect parameters changing during a negative energy balance of dairy cows and milk production-related traits [11–13]. When applied to high density marker data, the SMA approach usually has a massive multiple testing problem, which, when accounted for properly, substantially decreases the power to detect true genetic effects. Another shortcoming of the SMA approach is that it ignores the fact that genes may be represented by several markers and so the effect of a gene may be split up into several marker effects, each of which might not be large enough to pass the significance threshold. Therefore, especially in human genetics, researchers have come forward with gene-based association approaches aiming to overcome the limitations of the SMA [14–19].

In general, the main idea of a gene-based approach is to test each gene instead of each SNP separately by summarizing all SNP-effects annotated to a gene together to one main gene-effect. Here, one main challenge has been how to summarize the SNP-effects reasonably in order to obtain an efficient gene-level test statistic. To date, a number of methods has been proposed ranging from simple gene-level statistics like the minimum p-value approach [18] to complex statistics, which may account for the linkage disequilibrium (LD) structure or even integrate functional information of the SNPs [16,17,20,21]. For our analysis, we decided to employ the gene-based score test (GBST) adapted from Pan [21]. This approach accounts for the LD structure of the SNPs in a gene and the gene size measured by the number of SNPs, which in turn prevents the inflation of type I error.

In the following, we present the results of the GBST applied to the three key metabolites: (1) non-esterified fatty acids (NEFA), (2) beta-hydroxybutyrate (BHBA) and (3) glucose. We further use the significant genes to detect metabolic pathways potentially affecting these traits to gain understanding of their biological backgrounds. To this end, we adapt the methods commonly used for the analysis of gene expression profiles and gene sets in microarray data experiments, termed gene-set enrichment analysis (GSEA) [22]. More precisely, we employ the permutation-based weighted Kolmogorov Smirnov Test (WKST) by Subramanian et al. [23] and the Wilcoxon Rank Sum Test (WRST) [22] for the identification of pathways, which have been reported to be more successful than other approaches [22,24].

As a result, we found several biologically sensible genes and pathways associated with candidate metabolites during the transition period, which are essential for the adaptation of dairy cows. We further identified the three pathways involved in the metabolism of lipids and steroids, having a joint impact on all of our phenotypes. This may be regarded as evidence for the genetic basis for the adaptation performance of dairy cows and, at the same time, reveals its complexity.

## Results

### Analysis Overview

In order to assess the genetic characteristics of the metabolic adaptation of dairy cows during calving and lactation, we examined the three metabolites NEFA, BHBA and glucose at three critical points of time: 3 weeks before calving (T1), 4 and 13 weeks after calving (T2 and T3, respectively). According to van Dorland et al. [6], Graber et al. [10] and Gross et al. [2,4,7], these metabolites are key factors for the metabolic status of dairy cows during their early lactation period.

NEFA and BHBA, both serving as energy sources, are negatively correlated with feed intake and the synthesis of glucose, which is an essential substrate for milk synthesis. In general, dairy cows exhibit increased concentrations of NEFA and BHBA during the transition period resulting in a higher risk for diseases such as ketosis or fatty liver. Hence, we are especially interested in finding genes and pathways that could be responsible for regulating the concentration of NEFA, BHBA and glucose. In particular, we wish to find pathways that are able to inhibit the production of NEFA and BHBA and, at the same time, stimulate the production of glucose during the transition period. To this end, we performed the GBST and GSEA using the measurements of NEFA, BHBA and glucose at T1, T2 and T3. We also considered the changes of the metabolites over time and thus used the ratio of their concentrations measured at the different points of time (T2/T1, T3/T1, and T3/T2) for each metabolite, respectively, as additional phenotypes.

### Gene-based Analysis and SMA

For all the three traits considered, the GBST found 99 significant associations with the false discovery rate (FDR) approach [25] and 46 with the Bonferroni-correction at a genome-wide FDR or significance level of 5%. Table 1 summarizes the number of significant genes for each of the three phenotypes. A detailed description of the discovered genes is listed in S1 Table and the Manhattan plots for all our results can be found in S1–S3 Figs. As a comparison, we also performed a simple SMA that identified only two significant genes (FDR = 5%), which were also detected by the GBST. In the following, all results are based on the GBST at a genome-wide FDR level of 5%.

**Table 1:** Number of significant genes with the GBST (FDR = 5%) for the three metabolites.

Time/ratio	NEFA	BHBA	glucose
**T1**	5	8	5
**T2**	5	10	3
**T3**	7	0	12
**T2/T1**	2	9	8
**T3/T1**	10	0	3
**T3/T2**	9	2	1
**Sum**	38	29	32

First, we concentrate on the analysis of the results for the metabolite NEFA. Here, we found several genes on chromosome 3 and 13 affecting the concentration of NEFA during the ante- (T1) and post-partum (T3) period, respectively. However, these genes seem to have no statistical impact on the NEFA concentrations during the early lactation period with the highest metabolic load (T2). Moreover, we discover the gene SNAI2 (snail homolog (Drosophila)) on chromosome 14 to be significantly associated with the ratio of NEFA concentrations measured at T2 and T1 (T2/T1, *p = 6*.*28×10* ^*-7*^) as well as during T2 (*p = 5*.*08×10* ^*-8*^). This strongly supports the view that SNAI2 is important for the regulation of this metabolite during lactation and hence has an effect on the adaption of dairy cows. Also noticeable are the genes UGT2B15 (UDP glucuronosyltransferase 2 family, polypeptide B15) and MGC152010 (UDP glucuronosyltransferase 2 family) associated with the ratio of NEFA (T3/T1) with p-values of *p = 1*.*11×10* ^*-15*^ and *p = 1*.*27×10* ^*-11*^, respectively. This indicates that the regulation of the metabolic status of dairy cows before and after the onset of lactation is indeed different, even though usually the metabolic load is negligible at these times (T1 and T3).

In a similar fashion, we also identified several significant genes (i.a. DNAJC30 (DnaJ (Hsp40) homolog, subfamily C, member 30) and WBSCR22 (Williams Beuren syndrome chromosome region 22)) on chromosome 25 associated with the ratio of BHBA (T2/T1) with p-values smaller than *1*.*69×10* ^*-6*^. Interestingly, these genes also seem to affect the BHBA concentration strongly (all p-values < *9*.*22×10* ^*-10*^) during the early lactation period with the highest metabolic load (T2) but not 3 weeks before or 13 weeks after calving. Moreover, further investigations demonstrated that cows carrying both a minor allele at the SNP annotated to DNAJC30 and only major alleles at the three SNPs in WBSCR22 tend to have higher BHBA concentrations during the transition period (1.53 mmol/L on average) than cows with the opposite genetic characteristics (1.09 mmol/L on average). A Welch two sample t-test comparing the two different groups of cows yielded a p-value of *p = 0*.*044*.

Finally, we focused on the analysis of the phenotype glucose, an essential metabolite for the synthesis of milk during lactation. Here, the gene UEVLD (UEV and lactate/malate dehydrogenase domains) seems to play an important role in the regulation of the glucose concentration during lactation (T2, T2/T1). Similar to the results of BHBA, we investigated the 15 SNPs annotated to this gene. Among these SNPs, we identified 7 markers interacting with each other. Cows that carry only major alleles at these loci have a relatively high concentration of glucose in their blood at T2 (3.3 mmol/L on average, other cows: 3.0 mmol/L on average). Comparing the glucose concentration of these cows with the others yielded a p-value of *p = 3*.*3×10* ^*-7*^ at T2, but only *p = 0*.*01* and *p = 0*.*002* at T1 and T3. Even though the differences are significant at all three points in time, the effect appears to be highest during the early lactation period (T2, week 4 post-partum).

### Pathway Analysis

For the pathway analysis, we used the permutation-based weighted Kolmogorov Smirnov Test (WKST) by Subramanian et al. [23] as well as the Wilcoxon Rank Sum Test (WRST) [22] with *N* = 10,000 permutations. Note that, from a statistical point of view, the tests were performed in a two-step framework (see Methods Section), in which the results of the GBST were used as input information for the WKST and WRST. By doing so, we were able to ignore the uncertainty resulting from the estimation of the p-values for the GBST, which in turn could increase the uncertainty in the estimation of the p-values for the WKST and WRST. In order to account for this, we will only use the empirical p-values obtained by the WKST and WRST in the following analysis to rank the pathways according to their importance, but will not look at their significance. The aim is then to identify biologically and physiologically sensible pathways among the five top ranked pathways with the smallest p-values for each of the three phenotypes.

S2 and S3 Tables show the five top ranked pathways for each of the three phenotypes and points in time with the WKST and WRST, respectively. Due to the huge amount of results, we predominantly concentrated on pathways actively influencing the three phenotypes at T2 and T2/T1. Table 2 lists all the pathways ranked at least fifth by both WKST as well as WRST. Among the 20 phenotype-to-pathway associations, we found many associations to be biologically and physiologically sensible. We were further able to connect most of our findings to other studies (see Table 2).

**Table 2:** Phenotype-pathway associations that were at least fifth ranked by the WKST as well as the WRST, and references supporting the corresponding association if known.

Phenotype	Time	Pathways	Literature
NEFA	T2	Histidine metabolism	[26]
		Sulfur metabolism	
	T2/T1	Glycerolipid metabolism	[27]
		Glycerophospholipid metabolism	[27]
		Taurine and hypotaurine metabolism	
BHBA	T2	Retinol metabolism	[28]
		Tyrosine metabolism	
		Inositol phosphate metabolism	
		Steroid hormone biosynthesis	
	T2/T1	Synthesis and degradation of ketone bodies	[29]
		Tryptophan metabolism	
		Inositol phosphate metabolism	
glucose	T2	Steroid biosynthesis	[30]
		Other glycan degradation	
		Fatty acid elongation	
		Ether lipid metabolism	
	T2/T1	Ether lipid metabolism	
		Starch and sucrose metabolism	[29]
		Steroid hormone biosynthesis	[30]
		Glycerophospholipid metabolism	

On the one hand, according to our expectations, the pathway involved in the synthesis and degradation of ketone bodies is significantly associated with the ratio of the ketone body BHBA as well as the pathway for the metabolism of starch and sucrose with glucose. On the other hand, we were not able to establish a significant association of the galactose metabolism with glucose during lactation. This association was only significant 13 weeks after calving (T3, compare S2 Table).

Finally, we concentrate on the joint analysis of the three metabolites to discover pathways involved in the regulation of all three phenotypes and thus important for the metabolic adaptation of dairy cows (see Methods Section). Table 3 lists the 5 top ranked pathways resulting from the mentioned analysis (only for T2 and T2/T1, for the complete results please refer to the supporting information).

**Table 3:** The 5 pathways with the smallest p-values according to the WKST and WRST for the joined analysis of the NEFA, BHBA and glucose measurements at T2 and T2/T1.

Time	Rank	WKST	P-Value (WKST)	WRST	P-Value (WRST)
**T2**	**1**	Steroid hormone biosynthesis	0.0018	Steroid hormone biosynthesis	0.0040
**T2**	**2**	Retinol metabolism	0.0063	Other glycan degradation	0.0199
**T2**	**3**	Drug metabolism—other enzymes	0.0115	Drug metabolism—cytochrome P450	0.0222
**T2**	**4**	Starch and sucrose metabolism	0.0124	Retinol metabolism	0.0223
**T2**	**5**	Other glycan degradation	0.0174	Starch and sucrose metabolism	0.0242
**T2/T1**	**1**	Ether lipid metabolism	0.0030	Glycerophospholipid metabolism	0
**T2/T1**	**2**	Glycerophospholipid metabolism	0.0034	Ether lipid metabolism	5.00E-04
**T2/T1**	**3**	Other glycan degradation	0.0061	Nitrogen metabolism	0.0028
**T2/T1**	**4**	Tyrosine metabolism	0.0113	Tyrosine metabolism	0.0151
**T2/T1**	**5**	Nitrogen metabolism	0.0144	Other glycan degradation	0.0174

**Fig 1:**
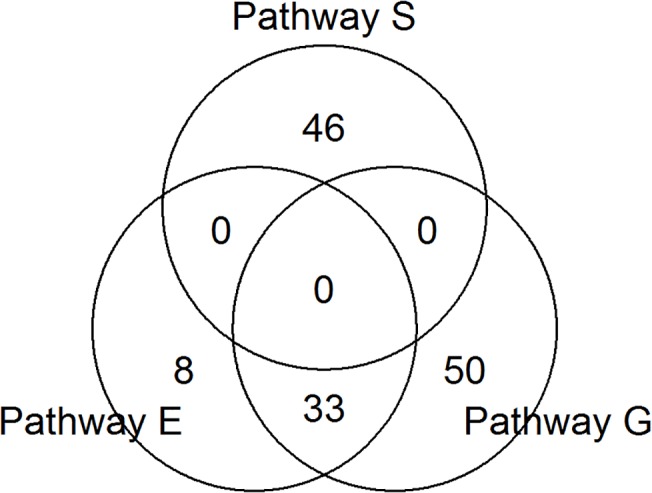
Venn-diagram for the number of genes annotated to the three pathways steroid hormone biosynthesis (S), ether lipid metabolism (E) and glycerophospholipid metabolism (G).

Interestingly, we found that the two pathways involved in the metabolism of ether lipids and glycerophospholipids are highly ranked by both the WKST as well as the WRST method. Moreover, both pathways demonstrate empirical p-values smaller than 0.005 at T2/T1. This finding agrees with the results of Klein et al. [31], who were able to establish a link between the glycerophosphocholine levels in milk and the susceptibility for ketosis in dairy cows during early lactation. As for T2, the pathway for steroid hormone biosynthesis is highly associated with the three metabolites, showing p-values of *p = 0*.*0018* and *p = 0*.*004* with the WKST and WRST, respectively. Fig. 1 illustrates the similarities of the three pathways and their number of genes. While the two lipid pathways share the majority of the genes involved, they seem to have no similarities with the steroid pathway.

## Discussion

Metabolic adaptation has been of great interest for dairy scientists during the last decades, but up to the present, little attention has been paid to its genetic aspects. In this study, we investigated the genetic factors influencing the metabolic adaptation of dairy cows during their transition period. In particular, we were interested in the two following questions:

(1) Do the differences in metabolic adaptation between cows have a genetic basis?
(2) If there is such a genetic basis, what genes and pathways are responsible for the metabolic adaptation?

As for the first question, our findings strongly support the idea that the metabolic adaption indeed has a genetic basis. Both the gene-based as well as the pathway analysis revealed many genes and pathways influencing the three metabolites, but only at certain points of time. For instance, the gene UEVLD appears to affect the phenotype glucose only in the early lactation, but not 4 weeks before or 13 weeks after calving. The opposite case can be observed from the relations between several genes on chromosome 3 and 13 and the phenotype NEFA.

With regard to the second question, we found several significant genes and pathways regulating the concentrations of NEFA, BHBA and glucose during the transition period. Three pathways with a number of SNPs detected (steroid hormone biosynthesis, ether lipid metabolism and glycerophospholipid metabolism) were found to jointly affect the key metabolites NEFA, BHBA and glucose. The genes distributing to the significance of the three pathways are involved in various sectors of the lipid metabolism. Especially, the repeated link to the cholesterol metabolism in dairy cows coping with elevated NEFA concentrations is obvious and was recently shown at a physiological level [32]. Interestingly, no SNPs were detected for genes, which are directly involved in the ketogenesis. However, the findings regarding associations to different phenotypes in BHBA concentrations are very probably an indirect result of the changes in NEFA concentrations. The availability or surplus of NEFA is a main factor determining the degree of BHBA synthesis, e.g. ketogenesis is regulated through NEFA plasma concentration. Similar to BHBA, glucose concentration cannot be directly connected to significant associations with SNPs in the presented genes and pathways. However, the occurrence of reduced glucose availability at high BHBA concentrations was recently demonstrated [33] and is presumably the reason for the low glucose concentration as a secondary effect of high NEFA concentration.

Generally, we were able to connect most of our findings to previous studies and hence confirm their plausibility. As an example, the associations of the glycerolipid and glycerophospholipid metabolism with the ratio of NEFA concentrations at T2 and T1 may be explained by the intense mobilization of lipids from tissue stores in the transition period resulting in a substantial release of NEFA into the blood stream [27]. Another interesting pathway associated with NEFA is the histidine metabolism (at T2). While there is no apparent connection between histidine and NEFA, Vanhatalo et al. [26] found that histidine significantly increases the milk and milk protein yield during lactation, but at the same time decreases its lactose and fat contents. Nevertheless, when interpreting these results, we have to take into account that even though the three considered metabolites are indeed indicators for the metabolic adaptation of dairy cows, they may not be fully indicative to the whole process of adaptation. Therefore, we suggest that the discovered genes and pathways should be viewed as potential candidates for closer investigation and validation from a biological perspective in future studies considering the complex endocrine and metabolic interactions.

In order to answer the two mentioned questions, we performed a GWAS using the GBST as suggested by Pan [21] followed by a GSEA for the identification of pathways. Even though the focus of this work was not methodological, our results demonstrated that using the GBST is more successful than the conventional SMA in identifying biologically sensible genes even with a relatively small samples size. For all of the considered traits, we discovered highly significant genes consisting of many SNPs interacting with each other. The SMA, however, missed most of these genes, since it is only designed to dissect single SNP-effects and has low power due to the massive multiple testing problem. Using the example of the Bonferroni-correction, Table 4 explains the apparent loss of power of the SMA compared to the GBST. By testing each single SNP separately, the SMA needs to test about 30 times more null hypotheses than the GBST resulting in much more conservative significance threshold per test.

**Table 4:** Comparison of the per test significance threshold between the SMA and GBST after adjustment with the Bonferroni-correction at a genome-wide level *α = 0*.*05*.

	*m* (# of SNPs/genes)	*α/m*	*-log*_*10*_ *(α/m)*
**SMA**	601,455 SNPs (all)	8.31×10^-8^	7.08
	231,712 SNPs (intragenic)	2.16×10^-7^	6.67
**GBST**	22,025 genes	2.27×10^-6^	5.64

The gene-based approach in combination with the pathway analysis is a well-established and commonly used method in human genetics. Here, a number of methods have been proposed and successfully employed to identify genes and pathways contributing to the development of complex human diseases (for some examples, see [15,16,19,20,34]). In animal genetics and breeding, researchers in general are still relying on simple SNP-based association studies. However, Peng et al. [15] as well as our study have shown that complex phenotypes are often affected by the joint action of many variants within a gene or even of many genes within a pathway. Hence, we believe that the use of the gene- and pathway-based approach in animal science is a promising tool to shed new light on the genetic complexity of common traits and deepen the understanding of their biological backgrounds.

## Material and Methods

### Phenotype Data

The data used in our analyses were obtained from a large on-farm study involving 232 dairy multiparous cows from different breed types (Brown Swiss, Holstein, Swiss Fleckvieh) housed at 64 farms (at least 2 cows per farm used in the trial, calving between November 2007 and April 2008, similar diet: grass and maize silage based feeding with additional concentrate, all cows under supervision of breeding associations) [35] and 50 Holstein dairy cows kept under controlled feeding conditions on an experimental farm (grass and maize silage based feeding with additional concentrate) [2]. In brief, blood samples were taken from cows which had a significant metabolic load in their previous early lactation as estimated by the fat:protein-ratio and milk fat content reflecting a tremendous body fat mobilization. Based on previous frequent measurements during the transition period, samples were taken at three critical stages of lactation: T1 = week 3 before expected calving (not lactating and no metabolic load); T2 = week 4 post-partum (lactating and high metabolic load), and T3 = week 13 after parturition (lactating and no metabolic load). Plasma concentrations of NEFA, BHBA and glucose were measured at T1, T2 and T3 using commercial kits as described by Graber et al. [35] and Gross et al. [2]. Table 5 presents the data summary for the three phenotypes and S4–S6 Figs. the corresponding histograms.

**Table 5:** Mean and standard deviation of the three metabolites NEFA, BHBA and glucose.

μ ± σ	NEFA log(mmol/L)	BHBA mmol/L	Glucose mmol/L
**T1**	4.269 ± 0.691	0.582 ± 0.266	3.719 ± 0.360
**T2**	5.624 ± 0.647	1.349 ± 1.081	3.146 ± 0.560
**T3**	4.643 ± 0.642	0.770 ± 0.352	3.702 ± 0.433

### Genotype Data

The 282 dairy cows were genotyped with the Illumina next-generation High-Density Bovine BeadChip. The resulting genotype dataset, consisting of 777,692 markers for 282 dairy cows, was then quality controlled and filtered with a SNP call rate of 95% and minor allele frequency (MAF) of 5%. After filtering and quality control, 601,455 SNPs for 282 animals remained.

**Fig 2:**
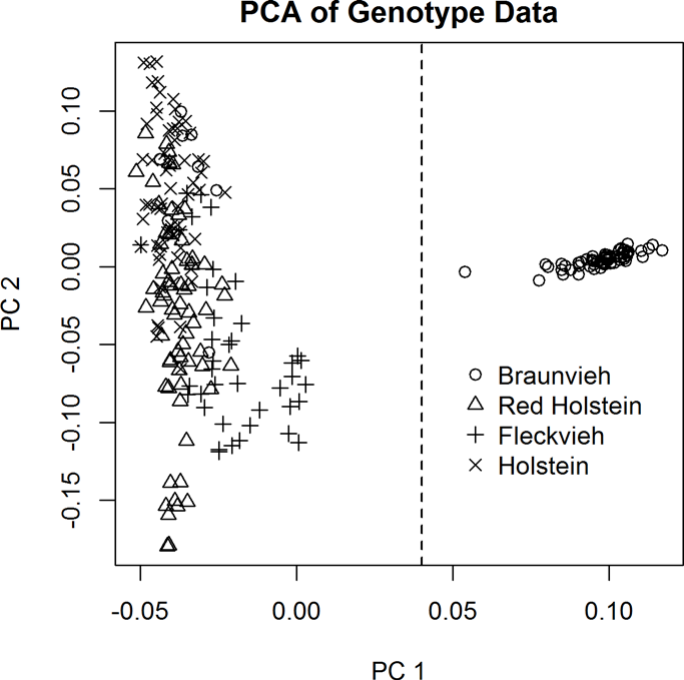
The two leading principal components of the analysis with the genotype data with 601,455 SNPs for 282 animals. The first and second principal components explain 8.7% and 1.4% of the total variation, respectively. The dashed line indicates the division of the cows into two groups.

To assess a possible stratification of the material, a principal component analysis (PCA) based on the genotypes was performed. Fig. 2 displays the first two principal components of the PCA. According to the results, we divided the 282 animals into two groups (Holstein, Red Holstein and Fleckvieh vs. Braunvieh). This grouping reflects that Swiss Fleckvieh historically was heavily interbred with Red Holstein. Within the two subgroups missing genotypes were imputed with the program BEAGLE (Version 3.3.2 [36]). After removing 38 cows with missing phenotypes, a final dataset with 178 animals in group 1 (**G1**) and 66 animals in group 2 (**G2**) was available for our analyses.

### Gene and Pathway Annotation

In order to perform the GBST and pathway analysis, we need a gene-annotation that allocates SNPs to genes and a pathway-annotation for genes to pathways. For the gene-annotation, we downloaded a list of all known genes from the Ensembl Genes database (Release 73, UMD3.1) for the species Bos taurus [37]. We allocated SNPs to genes according to the transcription-start and—end positions including exon, intron, UTR variants as well as SNPs up to 5kbp up- and downstream. The final gene-annotation includes 22,025 genes containing at least one intragenic SNP. For the pathway analysis, we retrieved a gene to pathway annotation from the Kyoto Encyclopedia of Genes and Genomes (KEGG) database [29]. We further matched the different gene identifiers from the Ensembl and KEGG database to obtain a final pathway-annotation for our dataset with 81 metabolic pathways containing at least 5 genes.

### Gene-based Mapping and Pathway Analysis

#### Statistical Model

Denote *y* the observed vector of phenotypes of *n* individuals and *X* an n×m matrix with values in {0, 1, 2} representing the genotypes for a gene *G* consisting of *m* SNPs. To test the association of the gene *G* to the phenotype *y*, we fitted a linear regression model

$$y=Z\theta +X\beta +\varepsilon ,\varepsilon \sim N(0,\sigma^{2}I_{n}),$$

where *Z* denotes the *n*×(*k*+1) matrix of covariates accounting for possible environmental factors or population structures, including the intercept and *θ* = (*θ*_1_,*θ*_2_,…,*θ*_*k*+1_)^*t*^, *β* = (*β*_1_,*β*_2_,…,*β*_*m*_)^*t*^ are the regression coefficients. Accordingly, the statistical problem is to test whether the phenotype *y* is influenced by any of the *m* SNPs, that is *H*_0_: *β* = 0. For the SMA approach, we performed a single marker regression and employed a simple t-test based on the same statistical model, but only for one SNP (*m* = 1) at a time.

#### Population Stratification and Environmental Factors

The principal component analysis based on the genotypes (see Fig. 2) revealed a substantial population structure within the group **G1** with 178 cows from the breeds Holstein, Red Holstein and Fleckvieh. To avoid inflation of the type I error [38], it is important to account for this stratification both in the gene-based as well as single marker analysis. We conducted a PCA for the group **G1** as to obtain covariates for the regression model and employed the Tracy-Widom test [38] to assess how many of the leading principal components contributed significantly to the population structure. As a result, the first 17 principal components were significant (*α* = 0.01) and thus were added as covariates to the linear regression model for the group **G1**. We chose a very conservative significant threshold in order to avoid overcorrection of the regression model, which would in turn mask possible true effects. The covariate matrix for the whole dataset (**G1**+**G2**) with *n* = 244 animals is then given by *Z* = (1_*n*_, *I*_*G*1_, *PC*_2_, …, *PC*_17_), where 1_*n*_ = (1, 1,…,1)^*t*^, (*I*_*G*1_)_*j*_ = 1 if animal *j* is in Group **G1** and 0 otherwise, and (*PC*_*i*_)_*j*_ is the value of the *i*-th principal component for animal *j* if it belongs to group **G1** and 0 otherwise for *j* = 1, 2,…,*n*.

The NEFA, BHBA and glucose measurements of the 244 cows are from two different studies, which might have different management systems. In order to account for this, we include an additional covariate *I*_*E*_ with (*I*_*E*_)_*j*_ = 1 if animal *j* is from the on-farm study and 0 otherwise. For comparison purposes, we perform the gene-based and pathway analysis twice, with the covariate matrix *Z* and Z* = (1_*n*_, *I*_*E*_, *I*_*G*1_, *PC*_1_, *PC*_2_,…, *PC*_17_). The results of the two analyses are similar, however, the analysis with the matrix *Z* yields smaller p-values. Therefore, in this study, we will present results based on the matrix *Z*.

#### Gene-based Score Test

For our gene-based analysis, we employed a modification of the Score Test, motivated by Pan [21]. To this end, denote *U* the score vector for the *m* SNPs adjusted for the covariates given by

$$U=\frac{1}{\hat{\sigma }}X^{t}(y-P_{Z}y),$$

where *P*_*Z*_ = *Z(Z* ^*t*^ *Z)* ^*−*1^ *Z* ^*t*^ and $\hat{\sigma }$ is the Maximum-Likelihood estimate for *σ* under *H*_0_. Then, the covariance matrix of *U*, also adjusted for the covariates *Z*, is

$$C={\left(X-P_{Z}X\right)}^{t}(X-P_{Z}X).$$

To test *H*_0_, Pan [21] suggests using the test statistic

$$SSU=U^{t}\text{Diag}{\left(C\right)}^{-1}U,$$

where Diag(*C*) is a diagonal matrix with only the diagonal elements of *C* as non-zero entries, instead of the standard score statistic *SSU΄* = *U* ^*t*^ *C* ^-1^ *U*. To calculate the distribution of the score statistic *SSU*, we used a *χ* ^2^-approximation by Zhang [39], while accounting for the fact that *σ* ^2^ was estimated by ${\hat{\sigma }}^{2}=\frac{1}{n}{\left(y-P_{Z}y\right)}^{t}(y-P_{Z}y)$.

According to Pan [21], the modified test statistic *SSU* is more powerful than *SSU’* for simulated genetic data. Freytag and Bickeboeller [40] also confirmed this finding by employing this method in the context of gene ranking. Using a simulation study, they compared this method to other summary statistics for genes and reported that the test based on the score statistic outperforms all other methods in many scenarios, in particular, when interactions between SNPs are present.

#### Pathway Analysis

Using the p-values obtained by the GBST, we performed a gene set enrichment analysis (GSEA) to identify metabolic pathways associated with the phenotypes. For this purpose, we adapted the weighted Kolmogorov-Smirnov Test (WKST) suggested by Subramanian et al. [23]. This method was shown to be superior compared to other gene set-level statistics by Hung et al. [22].

Denote *S* a given set of genes (e.g. a pathway) and *L* = {*g*_1_, *g*_2_,…,*g*_N_} a ranked list of all genes according to a ranking metric *r*(*g*_*j*_) = *r*_*j*_ with *r*_*1*_ ≥ *r*_*2*_ ≥…≥ *r*_*N*_. Importantly, the metric *r* should reflect the ‘importance’ of a gene for the phenotype under consideration.

In order to test the association of the pathway *S* and the considered phenotype according to the GSEA approach, we first calculate an Enrichment Score *ES* for this pathway and then permute the phenotypes to obtain its null distribution. To this end, we start with a running-sum *RS* = 0. We then walk down the list *L* (*i* = 1, 2,…,*N*) and increase *RS* by

$$\frac{r_{i}}{\sum_{g_{j}\in S}r_{j}}$$

if the gene *g*_*i*_ is contained in the pathway *S*, and decrease *RS* by

$$\frac{1}{N-N_{S}}$$

if the considered gene is not contained in the pathway *S*, where *N*_*S*_ = |*S*| is the number of genes in the pathway *S*. Finally, the Enrichment Score *ES* is defined by the maximum deviation of the running sum *RS* from zero. To assess the significance of *ES* while accounting for the correlation structure of the genes, we permuted the phenotypes and repeated the whole procedure 10,000 times for each pathway and phenotype. For comparison purposes, we also employed the permutation-based Wilcoxon Rank Sum Test (WRST) [22] based on the test statistic

$$RS=\sum_{g_{j}\in S}Rank_{L}(g_{j}).$$

In our analyses, we ranked our genes according to the p-values obtained by the GBST and set. r(g_j_) = −log_10_(p_j_) The analysis was performed for each metabolite and each points of time separately. Furthermore, in order to investigate whether there is a pathway that is able to regulate all three metabolites simultaneously, we also conducted a joint pathway analysis for the three metabolites. Here, we ranked the genes according to the product of the p-values obtained from the gene-based analysis with the three different phenotypes and set $r(g_{j})=-\log_{10}(p_{j}^{NEFA}p_{j}^{BHBA}p_{j}^{glu\cos e})$. This will especially raise the rank of genes exhibiting small p-values for all of the three metabolites. As a result, we hope to discover pathways involving in the regulation of all the three phenotypes and thus are important for the metabolic adaptation of dairy cows.

#### Multiple Hypothesis Testing

Both the SMA and the GBST require the testing of multiple hypotheses (SNPs or genes) simultaneously. Therefore, it is necessary to adjust the significance threshold of each single test properly in order to avoid an inflation of the genome-wide type I error rate. A commonly used, but very conservative method is the Bonferroni-correction. Here, the significance threshold for each single test *α’* is given by the genome-wide rate *α* (e.g. *α = 0*.*05*) divided by the number of hypotheses tested *m* ($\alpha '=\frac{\alpha }{m}$). By doing so, the probability for detecting at least one false positive signal, also termed as the familywise error rate (FWER), has an upper boundary determined by the level *α*. Another and more powerful method to control the type I error rate is the False Discovery Rate (FDR) approach by Benjamini and Hochberg [25]. As opposed to the Bonferroni-correction, the FDR approach aims to keep the expected proportion of false positives instead of the FWER below a certain level *q*. Thereby, the per test significance level *α’* is determined so that the equation $\frac{\alpha 'm}{R(\alpha ')}\leq q$ holds, where *R*(*α*΄) denotes the number of tests that were declared significant at the level *α’*.

## Supporting Information

S1 Fig Results of the GBST for NEFA. Manhatten plot of the GBST for the phenotype NEFA measured at T1 (1), T2 (2) and T3 (3) as well as the ratios. Each dot represents a gene. The dotted and dashed lines show the significance thresholds after the multiple testing correction according to Bonferroni and the FDR methods, respectively.
(JPEG)

S2 Fig Results of the GBST for BHBA. Manhatten plot of the GBST for the phenotype BHBA measured at T1 (1), T2 (2) and T3 (3) as well as the ratios. Each dot represents a gene. The dotted and dashed lines show the significance thresholds after the multiple testing correction according to Bonferroni and the FDR methods, respectively.
(JPEG)

S3 Fig Results of the GBST for glucose. Manhatten plot of the GBST for the phenotype glucose measured at T1 (1), T2 (2) and T3 (3) as well as the ratios. Each dot represents a gene. The dotted and dashed lines show the significance thresholds after the multiple testing correction according to Bonferroni and the FDR methods, respectively.
(JPEG)

S4 Fig Histograms of NEFA. Histograms of the phenotype NEFA measured at T1, T2 and T3. The different colors indicate the two different studies (grey = on-farm study).
(JPEG)

S5 Fig Histograms of BHBA. Histograms of the phenotype BHBA measured at T1, T2 and T3. The different colors indicate the two different studies (grey = on-farm study).
(JPEG)

S6 Fig Histograms of glucose. Histograms of the phenotype glucose measured at T1, T2 and T3. The different colors indicate the two different studies (grey = on-farm study).
(JPEG)

S1 Table Results of the GBST. Description of all significant genes (FDR < 5%) resulting from the gene-based score test (GBST) according to the Ensembl database.
(DOC)

S2 Table Results of the WKST. The five top ranked pathways according to the results of the weighted Kolmogorov Smirnov test (WKST).
(DOC)

S3 Table Results of the WRST. The five top ranked pathways according to the results of the Wilcoxon Rank Sum test (WRST).
(DOC)

S4 Table Results of the joint analysis with the WKST. The ten top ranked pathways according to the results of the joint analysis with all three metabolites with the weighted Kolmogorov Smirnov test (WKST).
(DOC)

S5 Table Results of the joint analysis with the WRST. The ten top ranked pathways according to the results of the joint analysis with all three metabolites with the Wilcoxon Rank Sum test (WRST).
(DOC)
