# Supplementary figures and images for: Gene-Based Mapping and Pathway Analysis of Metabolic Traits in Dairy Cows
Source: PLoS One. 2015 Mar 19;10(3):e0122325. doi: 10.1371/journal.pone.0122325 (PMC4366076; doi:10.1371/journal.pone.0122325)

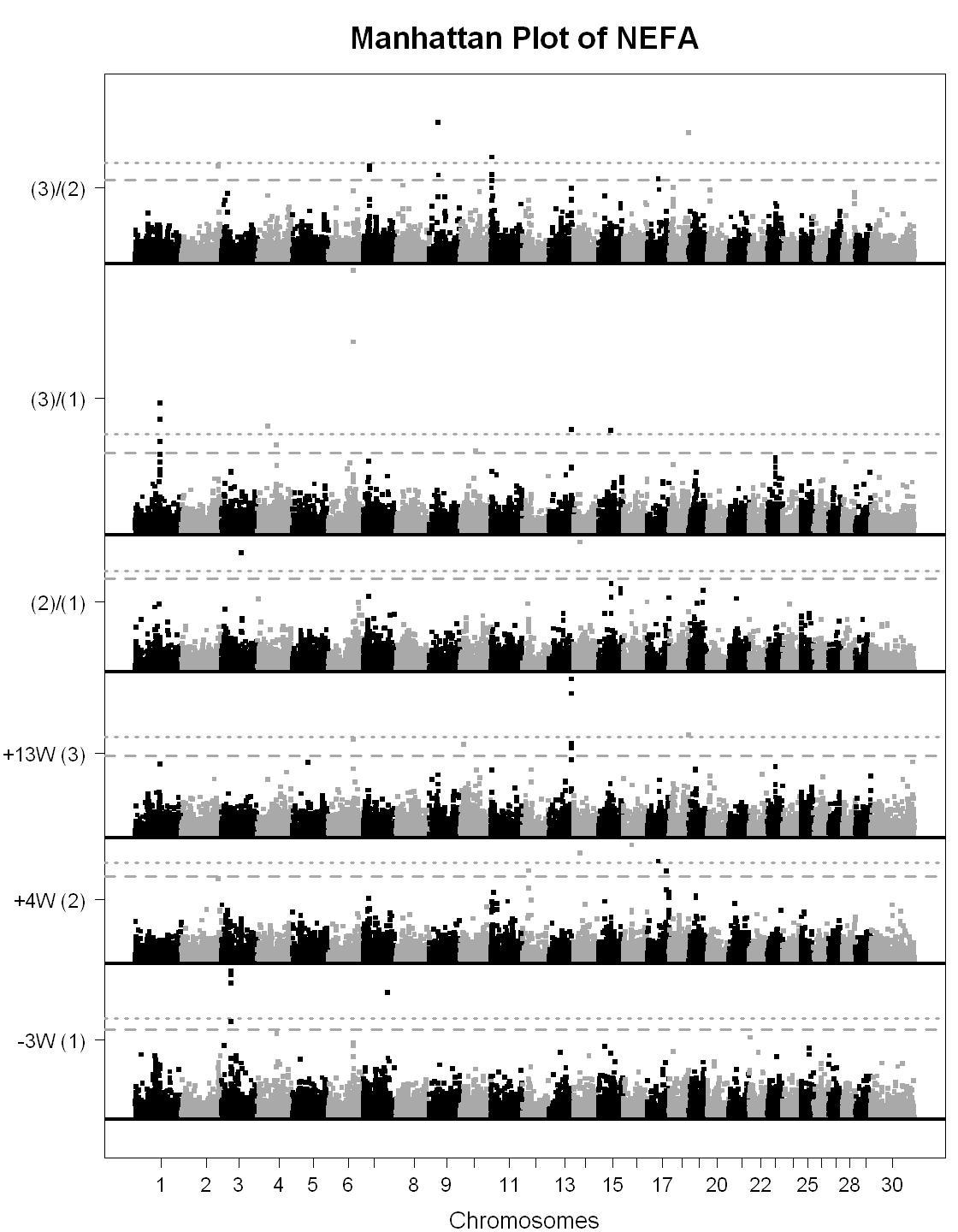

Supplement: S1 Fig — Manhatten plot of the GBST for the phenotype NEFA measured at T1 (1), T2 (2) and T3 (3) as well as the ratios. Each dot represents a gene. The dotted and dashed lines show the significance thresholds after the multiple testing correction according to Bonferroni and the FDR methods, respectively. (JPEG) [file pone.0122325.s001.jpeg]

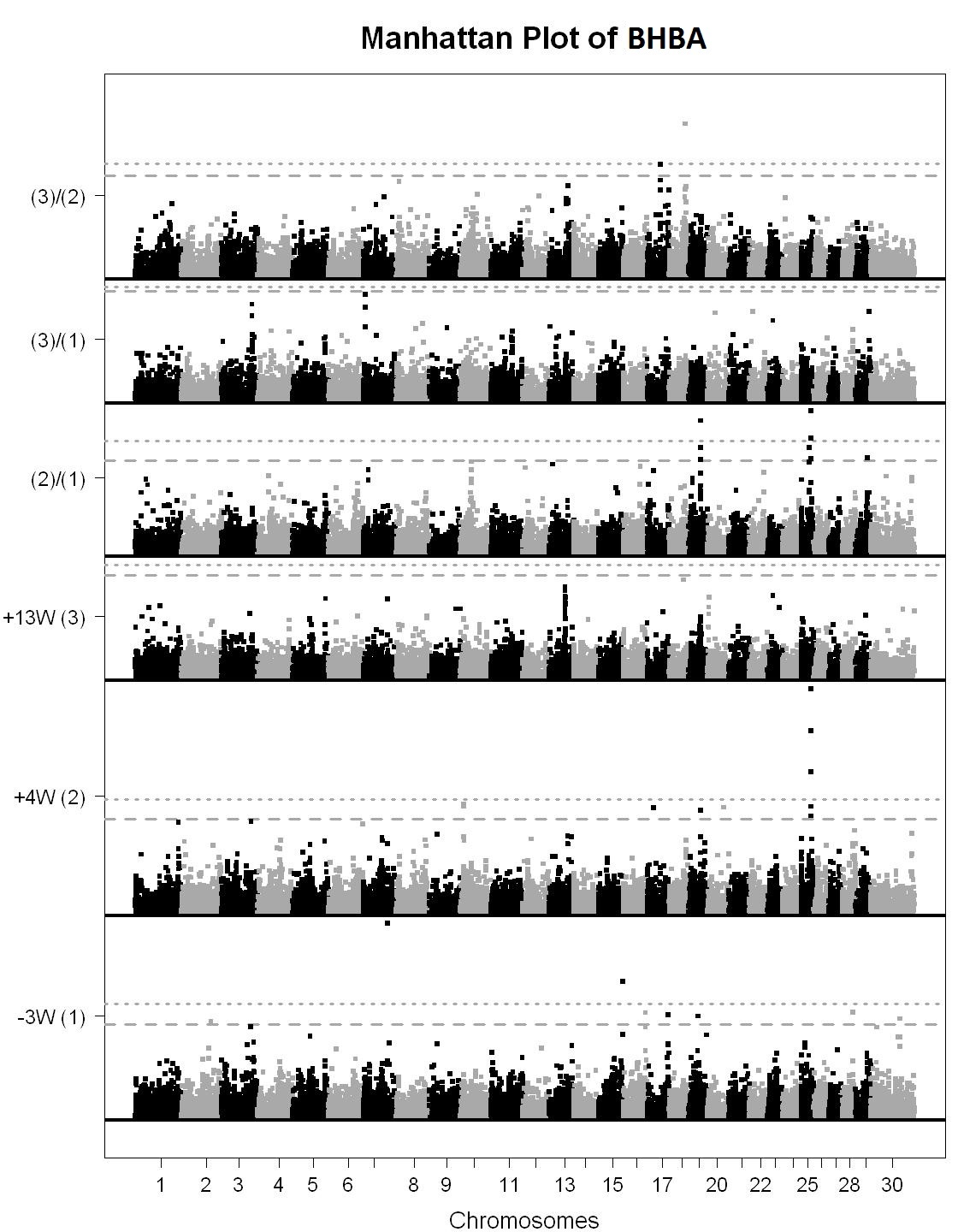

Supplement: S2 Fig — Manhatten plot of the GBST for the phenotype BHBA measured at T1 (1), T2 (2) and T3 (3) as well as the ratios. Each dot represents a gene. The dotted and dashed lines show the significance thresholds after the multiple testing correction according to Bonferroni and the FDR methods, respectively. (JPEG) [file pone.0122325.s002.jpeg]

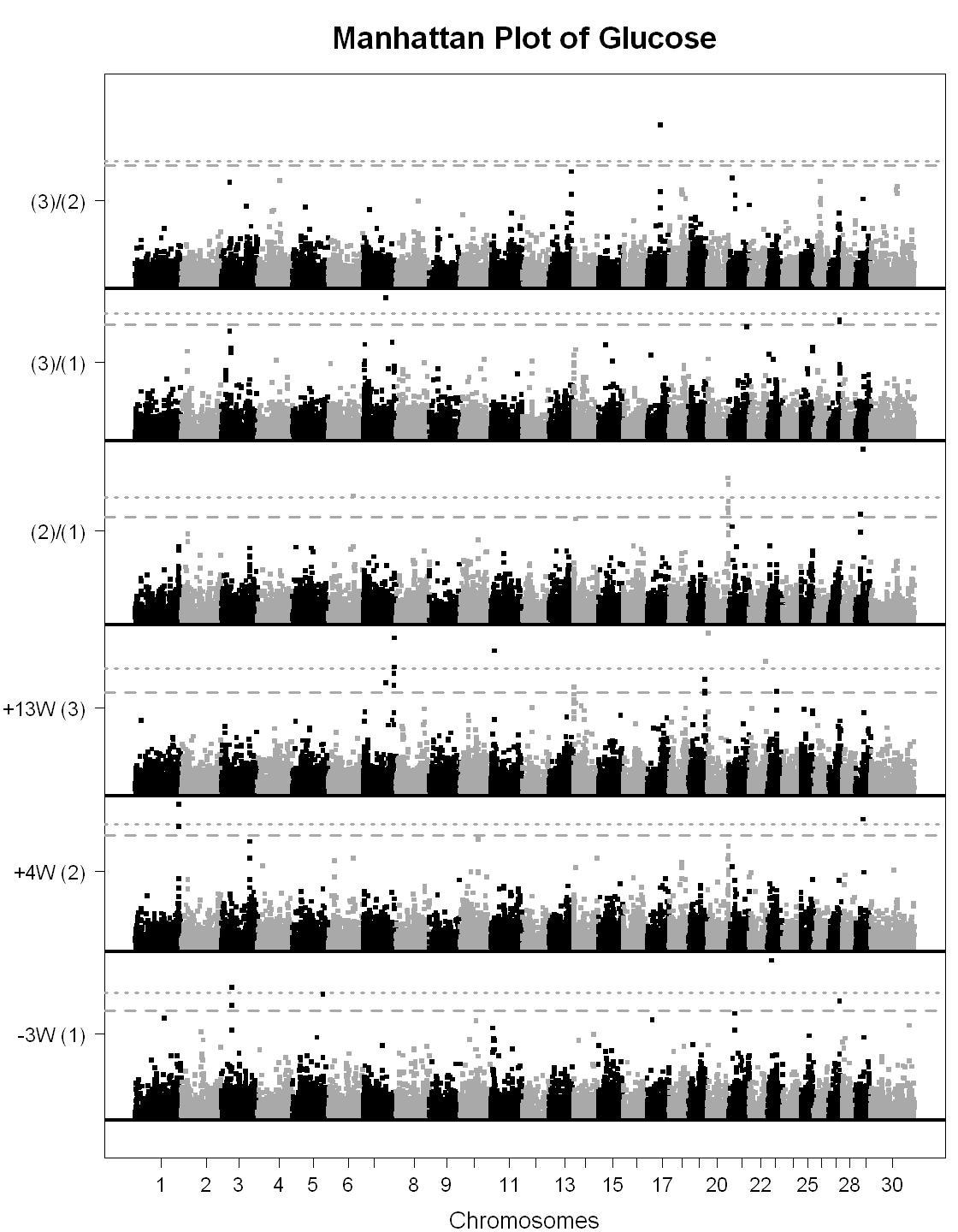

Supplement: S3 Fig — Manhatten plot of the GBST for the phenotype glucose measured at T1 (1), T2 (2) and T3 (3) as well as the ratios. Each dot represents a gene. The dotted and dashed lines show the significance thresholds after the multiple testing correction according to Bonferroni and the FDR methods, respectively. (JPEG) [file pone.0122325.s003.jpeg]

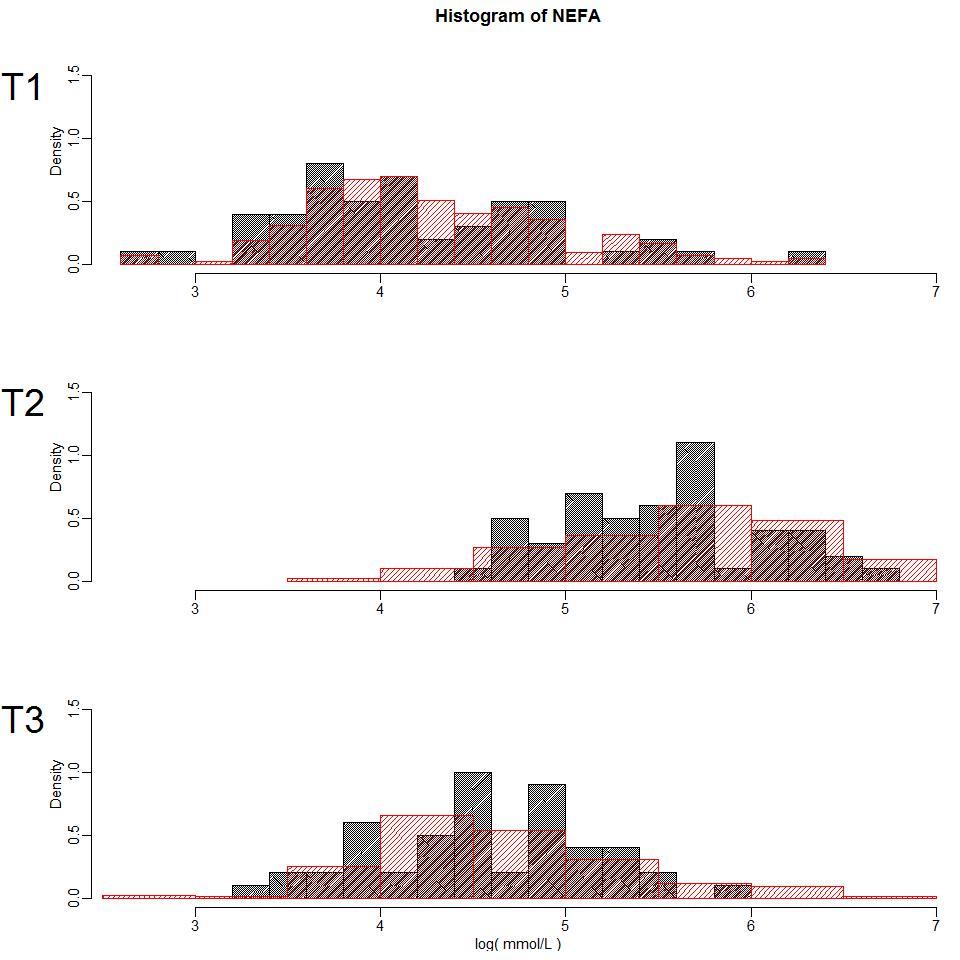

Supplement: S4 Fig — Histograms of the phenotype NEFA measured at T1, T2 and T3. The different colors indicate the two different studies (grey = on-farm study). (JPEG) [file pone.0122325.s004.jpeg]

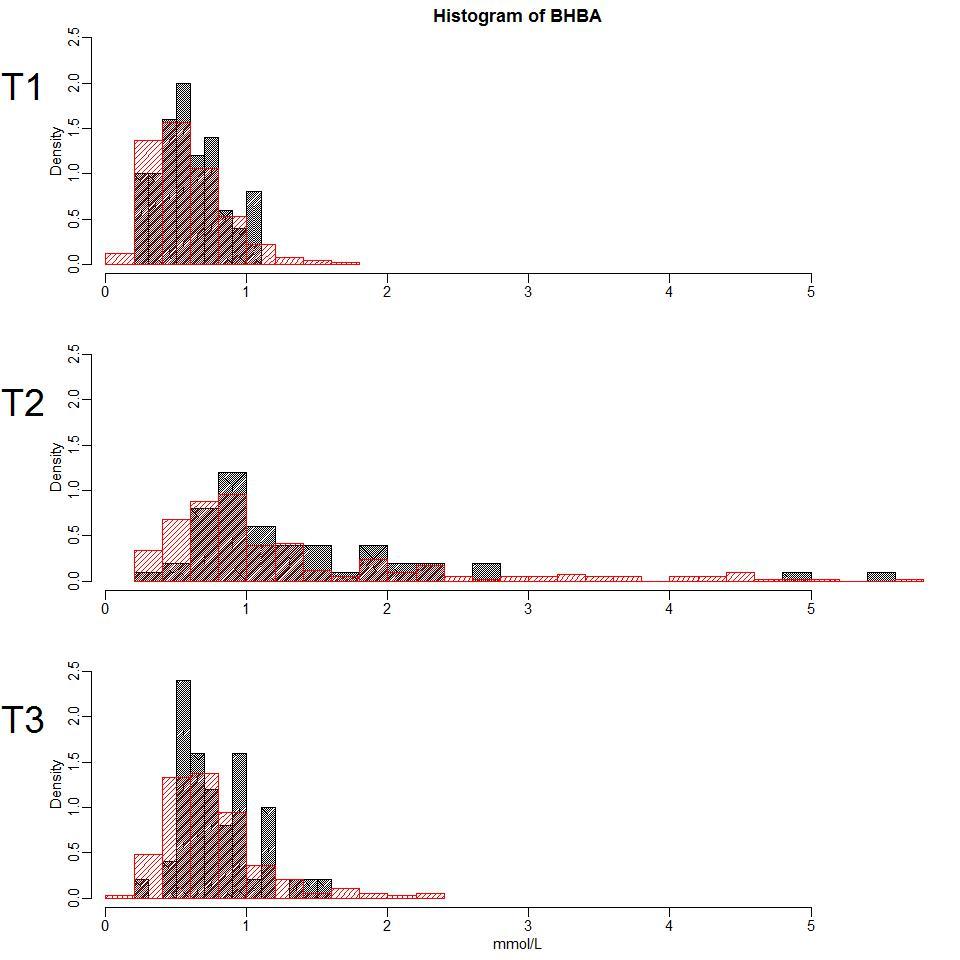

Supplement: S5 Fig — Histograms of the phenotype BHBA measured at T1, T2 and T3. The different colors indicate the two different studies (grey = on-farm study). (JPEG) [file pone.0122325.s005.jpeg]

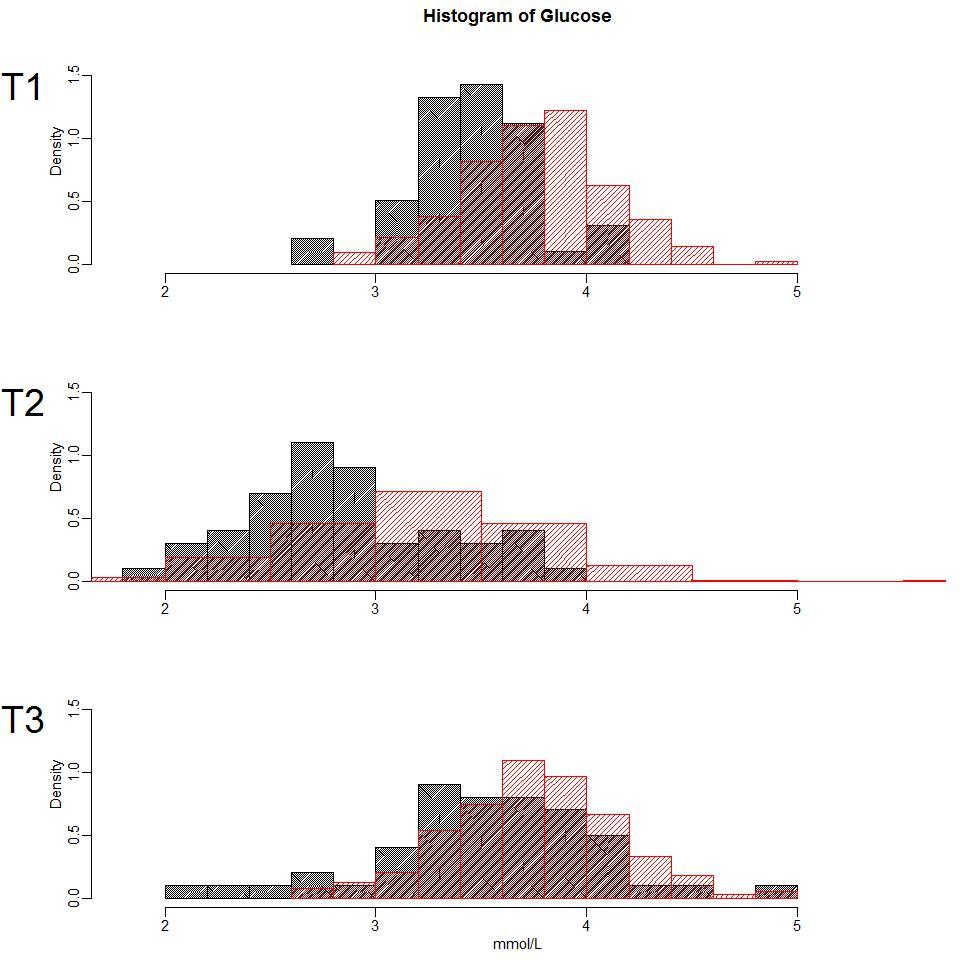

Supplement: S6 Fig — Histograms of the phenotype glucose measured at T1, T2 and T3. The different colors indicate the two different studies (grey = on-farm study). (JPEG) [file pone.0122325.s006.jpeg]
