# Supplementary material for: Gene-Based Mapping and Pathway Analysis of Metabolic Traits in Dairy Cows
Source: PLoS One. 2015 Mar 19;10(3):e0122325. doi: 10.1371/journal.pone.0122325 (PMC4366076; doi:10.1371/journal.pone.0122325)
Supplement: S1 Table — Description of all significant genes (FDR < 5%) resulting from the gene-based score test (GBST) according to the Ensembl database. (DOC) [file pone.0122325.s007.doc]

**Table S1. Results of the GBST.** Description of all significant genes (FDR < 5%) resulting from the gene-based score test (GBST) according to the Ensembl database.

| **Phenotype** | **Time** | **Ensemble Gene ID** | **Chromosome** | **Gene Size (# SNPs)** | **Gene Name** | **Description** | **P-Value** |
| --- | --- | --- | --- | --- | --- | --- | --- |
| NEFA | 1 | ENSBTAG00000030119 | 3 | 1 | Unknown | Unknown | 4.36E-09 |
| NEFA | 1 | ENSBTAG00000006466 | 3 | 11 | CD53 | CD53 molecule | 7.71E-09 |
| NEFA | 1 | ENSBTAG00000009888 | 3 | 5 | DRAM2 | DNA-damage regulated autophagy modulator 2 | 2.25E-08 |
| NEFA | 1 | ENSBTAG00000013856 | 7 | 37 | SSBP2 | single-stranded DNA binding protein 2 | 7.74E-08 |
| NEFA | 1 | ENSBTAG00000012659 | 3 | 3 | LRIF1 | ligand dependent nuclear receptor interacting factor 1 | 3.58E-06 |
| NEFA | 2 | ENSBTAG00000016185 | 16 | 25 | ENAH | enabled homolog (Drosophila) | 2.21E-07 |
| NEFA | 2 | ENSBTAG00000013227 | 14 | 1 | SNAI2 | snail homolog 2 (Drosophila) | 6.28E-07 |
| NEFA | 2 | ENSBTAG00000047595 | 17 | 21 | FSTL5 | follistatin-like 5 | 1.86E-06 |
| NEFA | 2 | ENSBTAG00000042372 | 12 | 1 | SNORA71 | Small nucleolar RNA SNORA71 | 6.29E-06 |
| NEFA | 2 | ENSBTAG00000043619 | 17 | 1 | U6 | U6 spliceosomal RNA | 6.67E-06 |
| NEFA | 3 | ENSBTAG00000030800 | 13 | 2 | WFDC10B | WAP Four-Disulfide Core Domain 10B | 1.11E-09 |
| NEFA | 3 | ENSBTAG00000030798 | 13 | 5 | WFDC11 | WAP four-disulfide core domain 11 | 7.57E-09 |
| NEFA | 3 | ENSBTAG00000003447 | 18 | 12 | ZNF551 | Zinc Finger Protein 551 | 1.71E-06 |
| NEFA | 3 | ENSBTAG00000003523 | 6 | 3 | UGT2B15 | UDP glucuronosyltransferase 2 family. polypeptide B15 | 3.15E-06 |
| NEFA | 3 | ENSBTAG00000017568 | 13 | 3 | WFDC13 | WAP four-disulfide core domain 13 | 5.30E-06 |
| NEFA | 3 | ENSBTAG00000019642 | 10 | 4 | ANKDD1A | ankyrin repeat and death domain containing 1A | 6.10E-06 |
| NEFA | 3 | ENSBTAG00000013928 | 13 | 3 | WFDC2 | WAP four-disulfide core domain 2 | 9.53E-06 |
| NEFA | 21 | ENSBTAG00000013227 | 14 | 1 | SNAI2 | snail homolog 2 (Drosophila) | 5.08E-08 |
| NEFA | 21 | ENSBTAG00000031082 | 3 | 5 | LOC781576 | mCG1723-like | 2.07E-07 |
| NEFA | 31 | ENSBTAG00000003523 | 6 | 3 | UGT2B15 | UDP glucuronosyltransferase 2 family. polypeptide B15 | 1.11E-15 |
| NEFA | 31 | ENSBTAG00000039647 | 6 | 5 | MGC152010 | UDP glucuronosyltransferase 2 family | 1.27E-11 |
| NEFA | 31 | ENSBTAG00000014459 | 1 | 1 | MAP6D1 | MAP6 domain containing 1 | 3.93E-08 |
| NEFA | 31 | ENSBTAG00000014449 | 1 | 1 | RPS6 | 40S Ribosomal Protein S6 | 3.31E-07 |
| NEFA | 31 | ENSBTAG00000018133 | 4 | 31 | SEMA3A | sema domain. immunoglobulin domain (Ig). short basic domain. secreted. (semaphorin) 3A | 8.05E-07 |
| NEFA | 31 | ENSBTAG00000030800 | 13 | 2 | WFDC10B | WAP four-disulfide core domain 10B | 1.20E-06 |
| NEFA | 31 | ENSBTAG00000045396 | 15 | 3 | U6 | U6 spliceosomal RNA | 1.42E-06 |
| NEFA | 31 | ENSBTAG00000038131 | 1 | 2 | ABCC5 | ATP-binding cassette. sub-family C (CFTR/MRP). member 5 | 5.85E-06 |
| NEFA | 31 | ENSBTAG00000020247 | 4 | 1 | ADCYAP1R1 | adenylate cyclase activating polypeptide 1 (pituitary) receptor type I | 9.66E-06 |
| NEFA | 31 | ENSBTAG00000013452 | 10 | 5 | AQP9 | aquaporin 9 | 2.08E-05 |
| NEFA | 32 | ENSBTAG00000009575 | 9 | 16 | C9H6orf170 | chromosome 9 open reading frame. human C6orf170 | 1.18E-08 |
| NEFA | 32 | ENSBTAG00000003447 | 18 | 12 | ZNF551 | zinc finger protein 551 | 4.65E-08 |
| NEFA | 32 | ENSBTAG00000048041 | 11 | 9 | ACTR1B | ARP1 actin-related protein 1 homolog B. centractin beta (yeast) | 1.08E-06 |
| NEFA | 32 | ENSBTAG00000013309 | 2 | 3 | SRSF4 | serine/arginine-rich splicing factor 4 | 3.53E-06 |
| NEFA | 32 | ENSBTAG00000031352 | 7 | 1 | ZNF554 | zinc finger protein 554 | 3.68E-06 |
| NEFA | 32 | ENSBTAG00000020446 | 7 | 5 | THOP1 | thimet oligopeptidase 1 | 5.93E-06 |
| NEFA | 32 | ENSBTAG00000005892 | 11 | 19 | ZAP70 | zeta-chain (TCR) associated protein kinase 70kDa | 1.14E-05 |
| NEFA | 32 | ENSBTAG00000044764 | 9 | 2 | SNORA25 | Small nucleolar RNA SNORA25 | 1.14E-05 |
| NEFA | 32 | ENSBTAG00000047595 | 17 | 21 | FSTL5 | follistatin-like 5 | 1.97E-05 |
| BHBA | 1 | ENSBTAG00000013880 | 7 | 16 | WWC1 | WW and C2 domain containing 1 | 2.29E-10 |
| BHBA | 1 | ENSBTAG00000002389 | 15 | 4 | MED19 | mediator complex subunit 19 | 1.70E-07 |
| BHBA | 1 | ENSBTAG00000046421 | 28 | 5 | SFTPD | surfactant protein D | 6.06E-06 |
| BHBA | 1 | ENSBTAG00000015086 | 16 | 9 | HSD11B1 | hydroxysteroid (11-beta) dehydrogenase 1 | 6.28E-06 |
| BHBA | 1 | ENSBTAG00000019146 | 17 | 35 | OSBP2 | oxysterol binding protein 2 | 7.69E-06 |
| BHBA | 1 | ENSBTAG00000031107 | 19 | 8 | HS3ST3A1 | heparan sulfate (glucosamine) 3-O-sulfotransferase 3A1 | 9.32E-06 |
| BHBA | 1 | ENSBTAG00000003418 | 30 | 5 | MSN | moesin | 1.23E-05 |
| BHBA | 1 | ENSBTAG00000047490 | 2 | 1 | Unknown | Unknown | 1.64E-05 |
| BHBA | 2 | ENSBTAG00000009022 | 25 | 1 | DNAJC30 | DnaJ (Hsp40) homolog. subfamily C. member 30 | 7.27E-12 |
| BHBA | 2 | ENSBTAG00000017067 | 25 | 3 | WBSCR22 | Williams Beuren syndrome chromosome region 22 | 9.22E-10 |
| BHBA | 2 | ENSBTAG00000000781 | 25 | 32 | HIP1 | huntingtin interacting protein 1 | 9.78E-08 |
| BHBA | 2 | ENSBTAG00000015155 | 10 | 15 | PTPLAD1 | protein tyrosine phosphatase-like A domain containing 1 | 4.15E-06 |
| BHBA | 2 | ENSBTAG00000015158 | 10 | 11 | C10H15orf44 | chromosome 10 open reading frame. human C15orf44 | 4.86E-06 |
| BHBA | 2 | ENSBTAG00000044466 | 25 | 2 | SCARNA20 | Small Cajal body specific RNA 20 | 5.07E-06 |
| BHBA | 2 | ENSBTAG00000044221 | 20 | 2 | SNORA9 | Small nucleolar RNA SNORA9 | 5.62E-06 |
| BHBA | 2 | ENSBTAG00000043885 | 17 | 2 | 7SK | 7SK RNA | 5.92E-06 |
| BHBA | 2 | ENSBTAG00000046907 | 19 | 2 | KRTAP4-12 | keratin associated protein 4-12 | 8.08E-06 |
| BHBA | 2 | ENSBTAG00000017075 | 25 | 5 | STX1A | syntaxin 1A (brain) | 1.51E-05 |
| BHBA | 21 | ENSBTAG00000009022 | 25 | 1 | DNAJC30 | DnaJ (Hsp40) homolog. subfamily C. member 30 | 7.44E-08 |
| BHBA | 21 | ENSBTAG00000046907 | 19 | 2 | KRTAP4-12 | keratin associated protein 4-12 | 2.24E-07 |
| BHBA | 21 | ENSBTAG00000017067 | 25 | 3 | WBSCR22 | Williams Beuren syndrome chromosome region 22 | 1.69E-06 |
| BHBA | 21 | ENSBTAG00000031551 | 25 | 1 | PRSS53 | protease. serine. 53 | 4.69E-06 |
| BHBA | 21 | ENSBTAG00000046075 | 19 | 4 | LOC777598 | keratin associated protein-like | 4.78E-06 |
| BHBA | 21 | ENSBTAG00000000405 | 25 | 2 | VKORC1 | vitamin K epoxide reductase complex. subunit 1 | 5.07E-06 |
| BHBA | 21 | ENSBTAG00000046860 | 29 | 4 | bta-mir-2885 | bta-mir-2885 | 1.55E-05 |
| BHBA | 21 | ENSBTAG00000000781 | 25 | 32 | HIP1 | huntingtin interacting protein 1 | 1.77E-05 |
| BHBA | 21 | ENSBTAG00000037943 | 19 | 1 | Unknown | Unknown | 1.94E-05 |
| BHBA | 32 | ENSBTAG00000013157 | 18 | 6 | NOVA2 | neuro-oncological ventral antigen 2 | 2.56E-08 |
| BHBA | 32 | ENSBTAG00000039316 | 17 | 3 | ZNF268 | zinc finger protein 268 | 2.63E-06 |
| Glucose | 1 | ENSBTAG00000048116 | 23 | 1 | Unknown | Unknown | 8.26E-08 |
| Glucose | 1 | ENSBTAG00000018893 | 3 | 3 | AHCYL1 | adenosylhomocysteinase-like 1 | 1.36E-06 |
| Glucose | 1 | ENSBTAG00000010490 | 5 | 8 | PEX5 | peroxisomal biogenesis factor 5 | 2.72E-06 |
| Glucose | 1 | ENSBTAG00000004015 | 27 | 8 | KAT6A | K(lysine) acetyltransferase 6A | 5.45E-06 |
| Glucose | 1 | ENSBTAG00000020964 | 3 | 4 | FAM40A | family with sequence similarity 40. member A | 8.87E-06 |
| Glucose | 2 | ENSBTAG00000030670 | 1 | 20 | PCNT | pericentrin | 2.90E-07 |
| Glucose | 2 | ENSBTAG00000013568 | 29 | 15 | UEVLD | UEV and lactate/malate dehyrogenase domains | 1.37E-06 |
| Glucose | 2 | ENSBTAG00000021984 | 1 | 4 | C21ORF58 | chromosome 21 open reading frame 58 | 2.96E-06 |
| Glucose | 3 | ENSBTAG00000017457 | 20 | 14 | STK10 | serine/threonine kinase 10 | 6.35E-08 |
| Glucose | 3 | ENSBTAG00000045413 | 7 | 7 | 7SK | 7SK RNA | 1.02E-07 |
| Glucose | 3 | ENSBTAG00000024058 | 11 | 2 | EGR4 | early growth response 4 | 3.81E-07 |
| Glucose | 3 | ENSBTAG00000001447 | 22 | 5 | HRH1 | histamine receptor H1 | 1.19E-06 |
| Glucose | 3 | ENSBTAG00000027727 | 7 | 34 | BT.94335 | Unknown | 2.07E-06 |
| Glucose | 3 | ENSBTAG00000021471 | 7 | 3 | GIN1 | gypsy retrotransposon integrase 1 | 4.04E-06 |
| Glucose | 3 | ENSBTAG00000047510 | 19 | 4 | LOC100302389 | uncharacterized LOC100302389 | 7.61E-06 |
| Glucose | 3 | ENSBTAG00000030286 | 7 | 7 | GABRA1 | gamma-aminobutyric acid (GABA) A receptor. alpha 1 | 1.09E-05 |
| Glucose | 3 | ENSBTAG00000001485 | 7 | 7 | PPIP5K2 | diphosphoinositol pentakisphosphate kinase 2 | 1.36E-05 |
| Glucose | 3 | ENSBTAG00000014642 | 14 | 3 | NAPRT1 | nicotinate phosphoribosyltransferase domain containing 1 | 1.63E-05 |
| Glucose | 3 | ENSBTAG00000011717 | 19 | 11 | SAP30BP | SAP30 binding protein | 2.48E-05 |
| Glucose | 3 | ENSBTAG00000040583 | 23 | 1 | LOC526989 | olfactory receptor. family 2. subfamily B. member 8 pseudogene-like | 2.66E-05 |
| Glucose | 21 | ENSBTAG00000013568 | 29 | 15 | UEVLD | UEV and lactate/malate dehyrogenase domains | 1.61E-08 |
| Glucose | 21 | ENSBTAG00000015843 | 20 | 5 | SLC6A3 | solute carrier family 6 (neurotransmitter transporter). member 3 | 3.10E-07 |
| Glucose | 21 | ENSBTAG00000039409 | 20 | 1 | Unknown | Unknown | 6.08E-07 |
| Glucose | 21 | ENSBTAG00000044890 | 6 | 2 | 5S_rRNA | 5S ribosomal RNA | 2.01E-06 |
| Glucose | 21 | ENSBTAG00000004629 | 20 | 3 | SLC9A3 | solute carrier family 9 (sodium/hydrogen exchanger). member 3 | 3.59E-06 |
| Glucose | 21 | ENSBTAG00000000944 | 20 | 5 | LPCAT1 | lysophosphatidylcholine acyltransferase 1 | 7.47E-06 |
| Glucose | 21 | ENSBTAG00000011405 | 20 | 14 | CEP72 | centrosomal protein 72kDa | 1.15E-05 |
| Glucose | 21 | ENSBTAG00000018188 | 29 | 2 | NDUFC2 | NADH dehydrogenase (ubiquinone) 1. subcomplex unknown. 2. 14.5kDa | 1.34E-05 |
| Glucose | 31 | ENSBTAG00000030286 | 7 | 7 | GABRA1 | gamma-aminobutyric acid (GABA) A receptor. alpha 1 | 4.55E-07 |
| Glucose | 31 | ENSBTAG00000004015 | 27 | 8 | KAT6A | K(lysine) acetyltransferase 6A | 4.55E-06 |
| Glucose | 31 | ENSBTAG00000001244 | 27 | 7 | PLAT | plasminogen activator. tissue | 5.81E-06 |
| Glucose | 32 | ENSBTAG00000039316 | 17 | 3 | ZNF268 | zinc finger protein 268 | 5.20E-08 |
