# Supplementary material for: Gene-Based Mapping and Pathway Analysis of Metabolic Traits in Dairy Cows
Source: PLoS One. 2015 Mar 19;10(3):e0122325. doi: 10.1371/journal.pone.0122325 (PMC4366076; doi:10.1371/journal.pone.0122325)
Supplement: S2 Table — The five top ranked pathways according to the results of the weighted Kolmogorov Smirnov test (WKST). (DOC) [file pone.0122325.s008.doc]

**Table S2.** **Results of the WKST.** The five top ranked pathways according to the results of the weighted Kolmogorov Smirnov test (WKST).

| **Phenotype** | **Time** | **KEGG Pathway ID** | **Size (# Genes)** | **P-Value** | **Description** |
| --- | --- | --- | --- | --- | --- |
| NEFA | 1 | path:bta00450 | 16 | 0,0142 | Selenocompound metabolism |
| NEFA | 1 | path:bta00480 | 47 | 0,0184 | Glutathione metabolism |
| NEFA | 1 | path:bta00601 | 26 | 0,021 | Glycosphingolipid biosynthesis - lacto and neolacto series |
| NEFA | 1 | path:bta00561 | 48 | 0,0245 | Glycerolipid metabolism |
| NEFA | 1 | path:bta00430 | 11 | 0,0355 | Taurine and hypotaurine metabolism |
| NEFA | 2 | path:bta00250 | 30 | 0,013 | Alanine, aspartate and glutamate metabolism |
| NEFA | 2 | path:bta00130 | 10 | 0,0173 | Ubiquinone and other terpenoid-quinone biosynthesis |
| NEFA | 2 | path:bta00340 | 22 | 0,0256 | Histidine metabolism |
| NEFA | 2 | path:bta00531 | 18 | 0,0502 | Glycosaminoglycan degradation |
| NEFA | 2 | path:bta00920 | 11 | 0,0539 | Sulfur metabolism |
| NEFA | 3 | path:bta00860 | 31 | 8,00E-04 | Porphyrin and chlorophyll metabolism |
| NEFA | 3 | path:bta00510 | 46 | 0,0757 | N-Glycan biosynthesis |
| NEFA | 3 | path:bta00740 | 11 | 0,0781 | Riboflavin metabolism |
| NEFA | 3 | path:bta00040 | 23 | 0,0804 | Pentose and glucuronate interconversions |
| NEFA | 3 | path:bta00511 | 19 | 0,0968 | Other glycan degradation |
| NEFA | 21 | path:bta00561 | 48 | 8,00E-04 | Glycerolipid metabolism |
| NEFA | 21 | path:bta00564 | 83 | 0,002 | Glycerophospholipid metabolism |
| NEFA | 21 | path:bta00983 | 33 | 0,0941 | Drug metabolism - other enzymes |
| NEFA | 21 | path:bta00250 | 30 | 0,0948 | Alanine, aspartate and glutamate metabolism |
| NEFA | 21 | path:bta00430 | 11 | 0,0952 | Taurine and hypotaurine metabolism |
| NEFA | 31 | path:bta00740 | 11 | 0,0027 | Riboflavin metabolism |
| NEFA | 31 | path:bta00860 | 31 | 0,0038 | Porphyrin and chlorophyll metabolism |
| NEFA | 31 | path:bta00561 | 48 | 0,0343 | Glycerolipid metabolism |
| NEFA | 31 | path:bta00531 | 18 | 0,0387 | Glycosaminoglycan degradation |
| NEFA | 31 | path:bta00053 | 17 | 0,0421 | Ascorbate and aldarate metabolism |
| NEFA | 32 | path:bta00140 | 46 | 0,0027 | Steroid hormone biosynthesis |
| NEFA | 32 | path:bta00100 | 17 | 0,0194 | Steroid biosynthesis |
| NEFA | 32 | path:bta00561 | 48 | 0,023 | Glycerolipid metabolism |
| NEFA | 32 | path:bta00053 | 17 | 0,0306 | Ascorbate and aldarate metabolism |
| NEFA | 32 | path:bta00830 | 51 | 0,031 | Retinol metabolism |
| BHBA | 1 | path:bta00500 | 41 | 0,0089 | Starch and sucrose metabolism |
| BHBA | 1 | path:bta00563 | 23 | 0,0145 | Glycosylphosphatidylinositol(GPI)-anchor biosynthesis |
| BHBA | 1 | path:bta00450 | 16 | 0,0163 | Selenocompound metabolism |
| BHBA | 1 | path:bta00052 | 27 | 0,0192 | Galactose metabolism |
| BHBA | 1 | path:bta00760 | 28 | 0,0274 | Nicotinate and nicotinamide metabolism |
| BHBA | 2 | path:bta00830 | 51 | 8,00E-04 | Retinol metabolism |
| BHBA | 2 | path:bta00350 | 33 | 0,0173 | Tyrosine metabolism |
| BHBA | 2 | path:bta00562 | 57 | 0,0195 | Inositol phosphate metabolism |
| BHBA | 2 | path:bta00140 | 46 | 0,0426 | Steroid hormone biosynthesis |
| BHBA | 2 | path:bta00053 | 17 | 0,0561 | Ascorbate and aldarate metabolism |
| BHBA | 3 | path:bta00565 | 41 | 0,0173 | Ether lipid metabolism |
| BHBA | 3 | path:bta00561 | 48 | 0,0237 | Glycerolipid metabolism |
| BHBA | 3 | path:bta00564 | 83 | 0,0242 | Glycerophospholipid metabolism |
| BHBA | 3 | path:bta00592 | 25 | 0,0811 | alpha-Linolenic acid metabolism |
| BHBA | 3 | path:bta00590 | 69 | 0,0896 | Arachidonic acid metabolism |
| BHBA | 21 | path:bta00072 | 7 | 0,0072 | Synthesis and degradation of ketone bodies |
| BHBA | 21 | path:bta00380 | 42 | 0,0101 | Tryptophan metabolism |
| BHBA | 21 | path:bta00510 | 46 | 0,0184 | N-Glycan biosynthesis |
| BHBA | 21 | path:bta00350 | 33 | 0,024 | Tyrosine metabolism |
| BHBA | 21 | path:bta00562 | 57 | 0,0294 | Inositol phosphate metabolism |
| BHBA | 31 | path:bta00601 | 26 | 0,0157 | Glycosphingolipid biosynthesis - lacto and neolacto series |
| BHBA | 31 | path:bta00533 | 15 | 0,0268 | Glycosaminoglycan biosynthesis - keratan sulfate |
| BHBA | 31 | path:bta00430 | 11 | 0,0327 | Taurine and hypotaurine metabolism |
| BHBA | 31 | path:bta00590 | 69 | 0,0361 | Arachidonic acid metabolism |
| BHBA | 31 | path:bta00592 | 25 | 0,0538 | alpha-Linolenic acid metabolism |
| BHBA | 32 | path:bta00630 | 23 | 0,0267 | Glyoxylate and dicarboxylate metabolism |
| BHBA | 32 | path:bta00020 | 30 | 0,105 | Citrate cycle (TCA cycle) |
| BHBA | 32 | path:bta00280 | 41 | 0,1108 | Valine, leucine and isoleucine degradation |
| BHBA | 32 | path:bta00564 | 83 | 0,1112 | Glycerophospholipid metabolism |
| BHBA | 32 | path:bta00330 | 51 | 0,1158 | Arginine and proline metabolism |
| Glucose | 1 | path:bta00630 | 23 | 0,0219 | Glyoxylate and dicarboxylate metabolism |
| Glucose | 1 | path:bta00510 | 46 | 0,0278 | N-Glycan biosynthesis |
| Glucose | 1 | path:bta00511 | 19 | 0,0363 | Other glycan degradation |
| Glucose | 1 | path:bta01230 | 67 | 0,0687 | Biosynthesis of amino acids |
| Glucose | 1 | path:bta00564 | 83 | 0,0702 | Glycerophospholipid metabolism |
| Glucose | 2 | path:bta00100 | 17 | 0,0038 | Steroid biosynthesis |
| Glucose | 2 | path:bta00511 | 19 | 0,006 | Other glycan degradation |
| Glucose | 2 | path:bta00062 | 24 | 0,0109 | Fatty acid elongation |
| Glucose | 2 | path:bta00140 | 46 | 0,0119 | Steroid hormone biosynthesis |
| Glucose | 2 | path:bta00565 | 41 | 0,0202 | Ether lipid metabolism |
| Glucose | 3 | path:bta00052 | 27 | 0,0017 | Galactose metabolism |
| Glucose | 3 | path:bta00140 | 46 | 0,0261 | Steroid hormone biosynthesis |
| Glucose | 3 | path:bta00500 | 41 | 0,0329 | Starch and sucrose metabolism |
| Glucose | 3 | path:bta00040 | 23 | 0,0396 | Pentose and glucuronate interconversions |
| Glucose | 3 | path:bta00592 | 25 | 0,0502 | alpha-Linolenic acid metabolism |
| Glucose | 21 | path:bta00565 | 41 | 0,0163 | Ether lipid metabolism |
| Glucose | 21 | path:bta00500 | 41 | 0,0204 | Starch and sucrose metabolism |
| Glucose | 21 | path:bta00140 | 46 | 0,0228 | Steroid hormone biosynthesis |
| Glucose | 21 | path:bta00564 | 83 | 0,0265 | Glycerophospholipid metabolism |
| Glucose | 21 | path:bta00591 | 35 | 0,0402 | Linoleic acid metabolism |
| Glucose | 31 | path:bta00190 | 119 | 0,0039 | Oxidative phosphorylation |
| Glucose | 31 | path:bta00511 | 19 | 0,0267 | Other glycan degradation |
| Glucose | 31 | path:bta00640 | 28 | 0,0562 | Propanoate metabolism |
| Glucose | 31 | path:bta00310 | 48 | 0,0735 | Lysine degradation |
| Glucose | 31 | path:bta00340 | 22 | 0,0773 | Histidine metabolism |
| Glucose | 32 | path:bta00270 | 31 | 0,0087 | Cysteine and methionine metabolism |
| Glucose | 32 | path:bta00620 | 38 | 0,0135 | Pyruvate metabolism |
| Glucose | 32 | path:bta00380 | 42 | 0,014 | Tryptophan metabolism |
| Glucose | 32 | path:bta00500 | 41 | 0,0184 | Starch and sucrose metabolism |
| Glucose | 32 | path:bta00071 | 41 | 0,0353 | Fatty acid degradation |
