# Supplementary material for: Gene-Based Mapping and Pathway Analysis of Metabolic Traits in Dairy Cows
Source: PLoS One. 2015 Mar 19;10(3):e0122325. doi: 10.1371/journal.pone.0122325 (PMC4366076; doi:10.1371/journal.pone.0122325)
Supplement: S3 Table — The five top ranked pathways according to the results of the Wilcoxon Rank Sum test (WRST). (DOC) [file pone.0122325.s009.doc]

**Table S3.** **Results of the WRST.** The five top ranked pathways according to the results of the Wilcoxon Rank Sum test (WRST).

| **Phenotype** | **Time** | **KEGG Pathway ID** | **Size (# Genes)** | **P-Value** | **Description** |
| --- | --- | --- | --- | --- | --- |
| NEFA | 1 | path:bta00561 | 48 | 0,0175 | Glycerolipid metabolism |
| NEFA | 1 | path:bta00480 | 47 | 0,0232 | Glutathione metabolism |
| NEFA | 1 | path:bta00450 | 16 | 0,0282 | Selenocompound metabolism |
| NEFA | 1 | path:bta00860 | 31 | 0,0345 | Porphyrin and chlorophyll metabolism |
| NEFA | 1 | path:bta00601 | 26 | 0,0363 | Glycosphingolipid biosynthesis - lacto and neolacto series |
| NEFA | 2 | path:bta00340 | 22 | 0,0071 | Histidine metabolism |
| NEFA | 2 | path:bta00982 | 49 | 0,0308 | Drug metabolism - cytochrome P450 |
| NEFA | 2 | path:bta00920 | 11 | 0,06 | Sulfur metabolism |
| NEFA | 2 | path:bta00790 | 12 | 0,0927 | Folate biosynthesis |
| NEFA | 2 | path:bta00140 | 46 | 0,0954 | Steroid hormone biosynthesis |
| NEFA | 3 | path:bta00860 | 31 | 0,0025 | Porphyrin and chlorophyll metabolism |
| NEFA | 3 | path:bta00512 | 27 | 0,0461 | Mucin type O-Glycan biosynthesis |
| NEFA | 3 | path:bta00830 | 51 | 0,0511 | Retinol metabolism |
| NEFA | 3 | path:bta00053 | 17 | 0,0697 | Ascorbate and aldarate metabolism |
| NEFA | 3 | path:bta00071 | 41 | 0,0843 | Fatty acid degradation |
| NEFA | 21 | path:bta00561 | 48 | 0 | Glycerolipid metabolism |
| NEFA | 21 | path:bta00564 | 83 | 6,00E-04 | Glycerophospholipid metabolism |
| NEFA | 21 | path:bta00430 | 11 | 0,0577 | Taurine and hypotaurine metabolism |
| NEFA | 21 | path:bta00511 | 19 | 0,0582 | Other glycan degradation |
| NEFA | 21 | path:bta00670 | 18 | 0,0753 | One carbon pool by folate |
| NEFA | 31 | path:bta00860 | 31 | 0,0025 | Porphyrin and chlorophyll metabolism |
| NEFA | 31 | path:bta00561 | 48 | 0,0373 | Glycerolipid metabolism |
| NEFA | 31 | path:bta00531 | 18 | 0,0424 | Glycosaminoglycan degradation |
| NEFA | 31 | path:bta00670 | 18 | 0,0684 | One carbon pool by folate |
| NEFA | 31 | path:bta00053 | 17 | 0,0783 | Ascorbate and aldarate metabolism |
| NEFA | 32 | path:bta00140 | 46 | 0,0094 | Steroid hormone biosynthesis |
| NEFA | 32 | path:bta00030 | 25 | 0,0246 | Pentose phosphate pathway |
| NEFA | 32 | path:bta00100 | 17 | 0,0347 | Steroid biosynthesis |
| NEFA | 32 | path:bta00040 | 23 | 0,0449 | Pentose and glucuronate interconversions |
| NEFA | 32 | path:bta00830 | 51 | 0,0462 | Retinol metabolism |
| BHBA | 1 | path:bta00500 | 41 | 0,0105 | Starch and sucrose metabolism |
| BHBA | 1 | path:bta00970 | 42 | 0,0111 | Aminoacyl-tRNA biosynthesis |
| BHBA | 1 | path:bta00100 | 17 | 0,0115 | Steroid biosynthesis |
| BHBA | 1 | path:bta00563 | 23 | 0,023 | Glycosylphosphatidylinositol(GPI)-anchor biosynthesis |
| BHBA | 1 | path:bta00450 | 16 | 0,0242 | Selenocompound metabolism |
| BHBA | 2 | path:bta00830 | 51 | 0,006 | Retinol metabolism |
| BHBA | 2 | path:bta00562 | 57 | 0,0173 | Inositol phosphate metabolism |
| BHBA | 2 | path:bta00400 | 5 | 0,0371 | Phenylalanine, tyrosine and tryptophan biosynthesis |
| BHBA | 2 | path:bta00140 | 46 | 0,0424 | Steroid hormone biosynthesis |
| BHBA | 2 | path:bta00350 | 33 | 0,0519 | Tyrosine metabolism |
| BHBA | 3 | path:bta00565 | 41 | 0,0125 | Ether lipid metabolism |
| BHBA | 3 | path:bta00561 | 48 | 0,0245 | Glycerolipid metabolism |
| BHBA | 3 | path:bta00564 | 83 | 0,0276 | Glycerophospholipid metabolism |
| BHBA | 3 | path:bta00120 | 15 | 0,0315 | Primary bile acid biosynthesis |
| BHBA | 3 | path:bta00140 | 46 | 0,069 | Steroid hormone biosynthesis |
| BHBA | 21 | path:bta00562 | 57 | 0,0161 | Inositol phosphate metabolism |
| BHBA | 21 | path:bta00380 | 42 | 0,0187 | Tryptophan metabolism |
| BHBA | 21 | path:bta00072 | 7 | 0,0232 | Synthesis and degradation of ketone bodies |
| BHBA | 21 | path:bta01230 | 67 | 0,0306 | Biosynthesis of amino acids |
| BHBA | 21 | path:bta00910 | 16 | 0,0376 | Nitrogen metabolism |
| BHBA | 31 | path:bta00590 | 69 | 0,0099 | Arachidonic acid metabolism |
| BHBA | 31 | path:bta00380 | 42 | 0,012 | Tryptophan metabolism |
| BHBA | 31 | path:bta00533 | 15 | 0,0171 | Glycosaminoglycan biosynthesis - keratan sulfate |
| BHBA | 31 | path:bta00601 | 26 | 0,0255 | Glycosphingolipid biosynthesis - lacto and neolacto series |
| BHBA | 31 | path:bta00410 | 28 | 0,0429 | beta-Alanine metabolism |
| BHBA | 32 | path:bta00564 | 83 | 0,0673 | Glycerophospholipid metabolism |
| BHBA | 32 | path:bta00562 | 57 | 0,098 | Inositol phosphate metabolism |
| BHBA | 32 | path:bta00512 | 27 | 0,1007 | Mucin type O-Glycan biosynthesis |
| BHBA | 32 | path:bta00630 | 23 | 0,1044 | Glyoxylate and dicarboxylate metabolism |
| BHBA | 32 | path:bta00983 | 33 | 0,1044 | Drug metabolism - other enzymes |
| Glucose | 1 | path:bta00510 | 46 | 0,0167 | N-Glycan biosynthesis |
| Glucose | 1 | path:bta00630 | 23 | 0,0205 | Glyoxylate and dicarboxylate metabolism |
| Glucose | 1 | path:bta00591 | 35 | 0,0277 | Linoleic acid metabolism |
| Glucose | 1 | path:bta00750 | 8 | 0,036 | Vitamin B6 metabolism |
| Glucose | 1 | path:bta00340 | 22 | 0,0438 | Histidine metabolism |
| Glucose | 2 | path:bta00100 | 17 | 0,0037 | Steroid biosynthesis |
| Glucose | 2 | path:bta00511 | 19 | 0,006 | Other glycan degradation |
| Glucose | 2 | path:bta00062 | 24 | 0,0099 | Fatty acid elongation |
| Glucose | 2 | path:bta00565 | 41 | 0,0111 | Ether lipid metabolism |
| Glucose | 2 | path:bta00052 | 27 | 0,0118 | Galactose metabolism |
| Glucose | 3 | path:bta00052 | 27 | 5,00E-04 | Galactose metabolism |
| Glucose | 3 | path:bta00500 | 41 | 0,0029 | Starch and sucrose metabolism |
| Glucose | 3 | path:bta00140 | 46 | 0,0215 | Steroid hormone biosynthesis |
| Glucose | 3 | path:bta00010 | 54 | 0,034 | Glycolysis / Gluconeogenesis |
| Glucose | 3 | path:bta00830 | 51 | 0,0382 | Retinol metabolism |
| Glucose | 21 | path:bta00565 | 41 | 0,0013 | Ether lipid metabolism |
| Glucose | 21 | path:bta00564 | 83 | 0,0051 | Glycerophospholipid metabolism |
| Glucose | 21 | path:bta00910 | 16 | 0,0085 | Nitrogen metabolism |
| Glucose | 21 | path:bta00500 | 41 | 0,019 | Starch and sucrose metabolism |
| Glucose | 21 | path:bta00140 | 46 | 0,0203 | Steroid hormone biosynthesis |
| Glucose | 31 | path:bta00190 | 119 | 0,0024 | Oxidative phosphorylation |
| Glucose | 31 | path:bta00020 | 30 | 0,037 | Citrate cycle (TCA cycle) |
| Glucose | 31 | path:bta00340 | 22 | 0,0536 | Histidine metabolism |
| Glucose | 31 | path:bta00280 | 41 | 0,059 | Valine, leucine and isoleucine degradation |
| Glucose | 31 | path:bta00511 | 19 | 0,062 | Other glycan degradation |
| Glucose | 32 | path:bta00270 | 31 | 0,0016 | Cysteine and methionine metabolism |
| Glucose | 32 | path:bta00620 | 38 | 0,013 | Pyruvate metabolism |
| Glucose | 32 | path:bta00630 | 23 | 0,0178 | Glyoxylate and dicarboxylate metabolism |
| Glucose | 32 | path:bta00500 | 41 | 0,0183 | Starch and sucrose metabolism |
| Glucose | 32 | path:bta00052 | 27 | 0,0257 | Galactose metabolism |
