# Supplementary material for: Gene-Based Mapping and Pathway Analysis of Metabolic Traits in Dairy Cows
Source: PLoS One. 2015 Mar 19;10(3):e0122325. doi: 10.1371/journal.pone.0122325 (PMC4366076; doi:10.1371/journal.pone.0122325)
Supplement: S4 Table — The ten top ranked pathways according to the results of the joint analysis with all three metabolites with the weighted Kolmogorov Smirnov test (WKST). (DOC) [file pone.0122325.s010.doc]

**Table S4.** **Results of the joint analysis with the WKST.** The ten top ranked pathways according to the results of the joint analysis with all three metabolites with the weighted Kolmogorov Smirnov test (WKST).

| **Phenotype** | **Time** | **KEGG Pathway ID** | **Size (# Genes)** | **P-Value** | **Description** |
| --- | --- | --- | --- | --- | --- |
| NEFA-BHBA-Glucose | 1 | path:bta01040 | 21 | 0,0058 | Biosynthesis of unsaturated fatty acids |
| NEFA-BHBA-Glucose | 1 | path:bta00980 | 50 | 0,0138 | Metabolism of xenobiotics by cytochrome P450 |
| NEFA-BHBA-Glucose | 1 | path:bta00564 | 83 | 0,0183 | Glycerophospholipid metabolism |
| NEFA-BHBA-Glucose | 1 | path:bta00790 | 12 | 0,0311 | Folate biosynthesis |
| NEFA-BHBA-Glucose | 1 | path:bta00450 | 16 | 0,0312 | Selenocompound metabolism |
| NEFA-BHBA-Glucose | 1 | path:bta00480 | 47 | 0,0351 | Glutathione metabolism |
| NEFA-BHBA-Glucose | 1 | path:bta00982 | 49 | 0,0552 | Drug metabolism - cytochrome P450 |
| NEFA-BHBA-Glucose | 1 | path:bta00100 | 17 | 0,0583 | Steroid biosynthesis |
| NEFA-BHBA-Glucose | 1 | path:bta00592 | 25 | 0,0592 | alpha-Linolenic acid metabolism |
| NEFA-BHBA-Glucose | 1 | path:bta00591 | 35 | 0,0783 | Linoleic acid metabolism |
| NEFA-BHBA-Glucose | 2 | path:bta00140 | 46 | 0,0018 | Steroid hormone biosynthesis |
| NEFA-BHBA-Glucose | 2 | path:bta00830 | 51 | 0,0063 | Retinol metabolism |
| NEFA-BHBA-Glucose | 2 | path:bta00983 | 33 | 0,0115 | Drug metabolism - other enzymes |
| NEFA-BHBA-Glucose | 2 | path:bta00500 | 41 | 0,0124 | Starch and sucrose metabolism |
| NEFA-BHBA-Glucose | 2 | path:bta00511 | 19 | 0,0174 | Other glycan degradation |
| NEFA-BHBA-Glucose | 2 | path:bta00564 | 83 | 0,022 | Glycerophospholipid metabolism |
| NEFA-BHBA-Glucose | 2 | path:bta00350 | 33 | 0,0357 | Tyrosine metabolism |
| NEFA-BHBA-Glucose | 2 | path:bta00910 | 16 | 0,038 | Nitrogen metabolism |
| NEFA-BHBA-Glucose | 2 | path:bta00860 | 31 | 0,0381 | Porphyrin and chlorophyll metabolism |
| NEFA-BHBA-Glucose | 2 | path:bta00982 | 49 | 0,0386 | Drug metabolism - cytochrome P450 |
| NEFA-BHBA-Glucose | 3 | path:bta00040 | 23 | 0,0138 | Pentose and glucuronate interconversions |
| NEFA-BHBA-Glucose | 3 | path:bta00860 | 31 | 0,018 | Porphyrin and chlorophyll metabolism |
| NEFA-BHBA-Glucose | 3 | path:bta00052 | 27 | 0,019 | Galactose metabolism |
| NEFA-BHBA-Glucose | 3 | path:bta00500 | 41 | 0,0333 | Starch and sucrose metabolism |
| NEFA-BHBA-Glucose | 3 | path:bta00592 | 25 | 0,0379 | alpha-Linolenic acid metabolism |
| NEFA-BHBA-Glucose | 3 | path:bta00053 | 17 | 0,0544 | Ascorbate and aldarate metabolism |
| NEFA-BHBA-Glucose | 3 | path:bta00140 | 46 | 0,0603 | Steroid hormone biosynthesis |
| NEFA-BHBA-Glucose | 3 | path:bta00511 | 19 | 0,0672 | Other glycan degradation |
| NEFA-BHBA-Glucose | 3 | path:bta00561 | 48 | 0,076 | Glycerolipid metabolism |
| NEFA-BHBA-Glucose | 3 | path:bta00565 | 41 | 0,094 | Ether lipid metabolism |
| NEFA-BHBA-Glucose | 21 | path:bta00565 | 41 | 0,003 | Ether lipid metabolism |
| NEFA-BHBA-Glucose | 21 | path:bta00564 | 83 | 0,0034 | Glycerophospholipid metabolism |
| NEFA-BHBA-Glucose | 21 | path:bta00511 | 19 | 0,0061 | Other glycan degradation |
| NEFA-BHBA-Glucose | 21 | path:bta00350 | 33 | 0,0113 | Tyrosine metabolism |
| NEFA-BHBA-Glucose | 21 | path:bta00910 | 16 | 0,0144 | Nitrogen metabolism |
| NEFA-BHBA-Glucose | 21 | path:bta00140 | 46 | 0,0327 | Steroid hormone biosynthesis |
| NEFA-BHBA-Glucose | 21 | path:bta00500 | 41 | 0,0652 | Starch and sucrose metabolism |
| NEFA-BHBA-Glucose | 21 | path:bta00030 | 25 | 0,0755 | Pentose phosphate pathway |
| NEFA-BHBA-Glucose | 21 | path:bta00052 | 27 | 0,0849 | Galactose metabolism |
| NEFA-BHBA-Glucose | 21 | path:bta00983 | 33 | 0,0876 | Drug metabolism - other enzymes |
| NEFA-BHBA-Glucose | 31 | path:bta00410 | 28 | 0,0084 | beta-Alanine metabolism |
| NEFA-BHBA-Glucose | 31 | path:bta00430 | 11 | 0,0135 | Taurine and hypotaurine metabolism |
| NEFA-BHBA-Glucose | 31 | path:bta00561 | 48 | 0,0223 | Glycerolipid metabolism |
| NEFA-BHBA-Glucose | 31 | path:bta00053 | 17 | 0,0327 | Ascorbate and aldarate metabolism |
| NEFA-BHBA-Glucose | 31 | path:bta00310 | 48 | 0,0354 | Lysine degradation |
| NEFA-BHBA-Glucose | 31 | path:bta00510 | 46 | 0,039 | N-Glycan biosynthesis |
| NEFA-BHBA-Glucose | 31 | path:bta00511 | 19 | 0,0714 | Other glycan degradation |
| NEFA-BHBA-Glucose | 31 | path:bta00380 | 42 | 0,0772 | Tryptophan metabolism |
| NEFA-BHBA-Glucose | 31 | path:bta01212 | 48 | 0,0831 | Fatty acid metabolism |
| NEFA-BHBA-Glucose | 31 | path:bta01040 | 21 | 0,0881 | Biosynthesis of unsaturated fatty acids |
| NEFA-BHBA-Glucose | 32 | path:bta00830 | 51 | 0,0314 | Retinol metabolism |
| NEFA-BHBA-Glucose | 32 | path:bta00380 | 42 | 0,0352 | Tryptophan metabolism |
| NEFA-BHBA-Glucose | 32 | path:bta00250 | 30 | 0,0483 | Alanine, aspartate and glutamate metabolism |
| NEFA-BHBA-Glucose | 32 | path:bta00564 | 83 | 0,0508 | Glycerophospholipid metabolism |
| NEFA-BHBA-Glucose | 32 | path:bta00983 | 33 | 0,0528 | Drug metabolism - other enzymes |
| NEFA-BHBA-Glucose | 32 | path:bta00310 | 48 | 0,0544 | Lysine degradation |
| NEFA-BHBA-Glucose | 32 | path:bta00140 | 46 | 0,066 | Steroid hormone biosynthesis |
| NEFA-BHBA-Glucose | 32 | path:bta00591 | 35 | 0,0675 | Linoleic acid metabolism |
| NEFA-BHBA-Glucose | 32 | path:bta00030 | 25 | 0,0736 | Pentose phosphate pathway |
| NEFA-BHBA-Glucose | 32 | path:bta00100 | 17 | 0,0846 | Steroid biosynthesis |
