# Supplementary material for: Gene-Based Mapping and Pathway Analysis of Metabolic Traits in Dairy Cows
Source: PLoS One. 2015 Mar 19;10(3):e0122325. doi: 10.1371/journal.pone.0122325 (PMC4366076; doi:10.1371/journal.pone.0122325)
Supplement: S5 Table — The ten top ranked pathways according to the results of the joint analysis with all three metabolites with the Wilcoxon Rank Sum test (WRST). (DOC) [file pone.0122325.s011.doc]

**Table S5.** **Results of the joint analysis with the WRST.** The ten top ranked pathways according to the results of the joint analysis with all three metabolites with the Wilcoxon Rank Sum test (WRST).

| **Phenotype** | **Time** | **KEGG Pathway ID** | **Size (# Genes)** | **P-Value** | **Description** |
| --- | --- | --- | --- | --- | --- |
| NEFA-BHBA-Glucose | 1 | path:bta01040 | 21 | 0,0123 | Biosynthesis of unsaturated fatty acids |
| NEFA-BHBA-Glucose | 1 | path:bta00450 | 16 | 0,0195 | Selenocompound metabolism |
| NEFA-BHBA-Glucose | 1 | path:bta00564 | 83 | 0,0352 | Glycerophospholipid metabolism |
| NEFA-BHBA-Glucose | 1 | path:bta00510 | 46 | 0,0477 | N-Glycan biosynthesis |
| NEFA-BHBA-Glucose | 1 | path:bta00120 | 15 | 0,0575 | Primary bile acid biosynthesis |
| NEFA-BHBA-Glucose | 1 | path:bta00910 | 16 | 0,0633 | Nitrogen metabolism |
| NEFA-BHBA-Glucose | 1 | path:bta00533 | 15 | 0,0801 | Glycosaminoglycan biosynthesis - keratan sulfate |
| NEFA-BHBA-Glucose | 1 | path:bta00790 | 12 | 0,0808 | Folate biosynthesis |
| NEFA-BHBA-Glucose | 1 | path:bta00500 | 41 | 0,1032 | Starch and sucrose metabolism |
| NEFA-BHBA-Glucose | 1 | path:bta00561 | 48 | 0,1063 | Glycerolipid metabolism |
| NEFA-BHBA-Glucose | 2 | path:bta00140 | 46 | 0,004 | Steroid hormone biosynthesis |
| NEFA-BHBA-Glucose | 2 | path:bta00511 | 19 | 0,0199 | Other glycan degradation |
| NEFA-BHBA-Glucose | 2 | path:bta00982 | 49 | 0,0222 | Drug metabolism - cytochrome P450 |
| NEFA-BHBA-Glucose | 2 | path:bta00830 | 51 | 0,0223 | Retinol metabolism |
| NEFA-BHBA-Glucose | 2 | path:bta00500 | 41 | 0,0242 | Starch and sucrose metabolism |
| NEFA-BHBA-Glucose | 2 | path:bta00983 | 33 | 0,0244 | Drug metabolism - other enzymes |
| NEFA-BHBA-Glucose | 2 | path:bta00250 | 30 | 0,04 | Alanine, aspartate and glutamate metabolism |
| NEFA-BHBA-Glucose | 2 | path:bta00564 | 83 | 0,043 | Glycerophospholipid metabolism |
| NEFA-BHBA-Glucose | 2 | path:bta00980 | 50 | 0,0597 | Metabolism of xenobiotics by cytochrome P450 |
| NEFA-BHBA-Glucose | 2 | path:bta00910 | 16 | 0,0713 | Nitrogen metabolism |
| NEFA-BHBA-Glucose | 3 | path:bta00140 | 46 | 0,0109 | Steroid hormone biosynthesis |
| NEFA-BHBA-Glucose | 3 | path:bta00052 | 27 | 0,0141 | Galactose metabolism |
| NEFA-BHBA-Glucose | 3 | path:bta00500 | 41 | 0,0176 | Starch and sucrose metabolism |
| NEFA-BHBA-Glucose | 3 | path:bta00860 | 31 | 0,021 | Porphyrin and chlorophyll metabolism |
| NEFA-BHBA-Glucose | 3 | path:bta00040 | 23 | 0,031 | Pentose and glucuronate interconversions |
| NEFA-BHBA-Glucose | 3 | path:bta00592 | 25 | 0,0313 | alpha-Linolenic acid metabolism |
| NEFA-BHBA-Glucose | 3 | path:bta00591 | 35 | 0,0318 | Linoleic acid metabolism |
| NEFA-BHBA-Glucose | 3 | path:bta00053 | 17 | 0,04 | Ascorbate and aldarate metabolism |
| NEFA-BHBA-Glucose | 3 | path:bta00010 | 54 | 0,047 | Glycolysis / Gluconeogenesis |
| NEFA-BHBA-Glucose | 3 | path:bta00590 | 69 | 0,067 | Arachidonic acid metabolism |
| NEFA-BHBA-Glucose | 21 | path:bta00564 | 83 | 0 | Glycerophospholipid metabolism |
| NEFA-BHBA-Glucose | 21 | path:bta00565 | 41 | 5,00E-04 | Ether lipid metabolism |
| NEFA-BHBA-Glucose | 21 | path:bta00910 | 16 | 0,0028 | Nitrogen metabolism |
| NEFA-BHBA-Glucose | 21 | path:bta00350 | 33 | 0,0151 | Tyrosine metabolism |
| NEFA-BHBA-Glucose | 21 | path:bta00511 | 19 | 0,0174 | Other glycan degradation |
| NEFA-BHBA-Glucose | 21 | path:bta00592 | 25 | 0,0259 | alpha-Linolenic acid metabolism |
| NEFA-BHBA-Glucose | 21 | path:bta00450 | 16 | 0,0453 | Selenocompound metabolism |
| NEFA-BHBA-Glucose | 21 | path:bta00591 | 35 | 0,0507 | Linoleic acid metabolism |
| NEFA-BHBA-Glucose | 21 | path:bta00561 | 48 | 0,0572 | Glycerolipid metabolism |
| NEFA-BHBA-Glucose | 21 | path:bta00140 | 46 | 0,0622 | Steroid hormone biosynthesis |
| NEFA-BHBA-Glucose | 31 | path:bta00430 | 11 | 0,0243 | Taurine and hypotaurine metabolism |
| NEFA-BHBA-Glucose | 31 | path:bta00410 | 28 | 0,0407 | beta-Alanine metabolism |
| NEFA-BHBA-Glucose | 31 | path:bta00310 | 48 | 0,041 | Lysine degradation |
| NEFA-BHBA-Glucose | 31 | path:bta00561 | 48 | 0,0417 | Glycerolipid metabolism |
| NEFA-BHBA-Glucose | 31 | path:bta00511 | 19 | 0,0521 | Other glycan degradation |
| NEFA-BHBA-Glucose | 31 | path:bta00601 | 26 | 0,0638 | Glycosphingolipid biosynthesis - lacto and neolacto series |
| NEFA-BHBA-Glucose | 31 | path:bta00533 | 15 | 0,0666 | Glycosaminoglycan biosynthesis - keratan sulfate |
| NEFA-BHBA-Glucose | 31 | path:bta00510 | 46 | 0,0774 | N-Glycan biosynthesis |
| NEFA-BHBA-Glucose | 31 | path:bta01040 | 21 | 0,0803 | Biosynthesis of unsaturated fatty acids |
| NEFA-BHBA-Glucose | 31 | path:bta00380 | 42 | 0,0919 | Tryptophan metabolism |
| NEFA-BHBA-Glucose | 32 | path:bta00591 | 35 | 0,0197 | Linoleic acid metabolism |
| NEFA-BHBA-Glucose | 32 | path:bta00830 | 51 | 0,0238 | Retinol metabolism |
| NEFA-BHBA-Glucose | 32 | path:bta00564 | 83 | 0,0248 | Glycerophospholipid metabolism |
| NEFA-BHBA-Glucose | 32 | path:bta01210 | 15 | 0,0335 | 2-Oxocarboxylic acid metabolism |
| NEFA-BHBA-Glucose | 32 | path:bta00140 | 46 | 0,0368 | Steroid hormone biosynthesis |
| NEFA-BHBA-Glucose | 32 | path:bta00380 | 42 | 0,0405 | Tryptophan metabolism |
| NEFA-BHBA-Glucose | 32 | path:bta00630 | 23 | 0,0682 | Glyoxylate and dicarboxylate metabolism |
| NEFA-BHBA-Glucose | 32 | path:bta00270 | 31 | 0,069 | Cysteine and methionine metabolism |
| NEFA-BHBA-Glucose | 32 | path:bta00100 | 17 | 0,0698 | Steroid biosynthesis |
| NEFA-BHBA-Glucose | 32 | path:bta00983 | 33 | 0,0878 | Drug metabolism - other enzymes |
